# Evolutionary Relatedness and Classification of Tumor-Inducing and Opine-Catabolic Plasmids in Three *Rhizobium rhizogenes* Strains Isolated from the Same Crown Gall Tumor

**DOI:** 10.1093/gbe/evz091

**Published:** 2019-04-27

**Authors:** Nemanja Kuzmanović, Joanna Puławska

**Affiliations:** 1 Julius Kühn-Institut, Federal Research Centre for Cultivated Plants (JKI), Institute for Epidemiology and Pathogen Diagnostics, Braunschweig, Germany; 2 Research Institute of Horticulture, Skierniewice, Poland

**Keywords:** agrobacteria, crown gall, tumor-inducing (Ti) plasmid, opine-catabolic (OC) plasmid, plasmid evolution, horizontal gene transfer

## Abstract

Plasmids play a crucial role in the ecology of agrobacteria. In this study, we sequenced tumor-inducing (Ti) and opine-catabolic (OC) plasmids in three *Rhizobium rhizogenes* (*Agrobacterium* biovar 2) strains isolated from the same crown gall tumor on “Colt” cherry rootstock and conducted comparative genomic analyses. Tumorigenic strains C5.7 and C6.5 carry nopaline-type Ti plasmids pTiC5.7/pTiC6.5, whereas the nonpathogenic strain Colt5.8 carries the nopaline-type OC plasmid pOC-Colt5.8. Overall, comparative genomic analysis indicated that pTiC5.7/pTiC6.5 and related Ti plasmids described before (pTiC58 and pTi-SAKURA) originate from a common ancestor, although they have diverged during evolution. On the other hand, plasmid pOC-Colt5.8 was most closely related to the well-known OC plasmid pAtK84b; however, analysis suggested that they had different evolutionary histories and seem to share a more distant common ancestor. Although the reconstruction of the evolutionary history of Ti and OC plasmids is still speculative, we hypothesized that nopaline-type Ti plasmid might originate from the nopaline-type OC plasmid. Our results suggested that OC plasmids are widespread and closely associated with crown gall tumors. Finally, we proposed a thorough scheme for classification of Ti and OC plasmids that is based on separate comparative analysis of each functional element of the plasmid studied.

## Introduction

Crown gall is a widespread bacterial disease that affects various fruit species, grapevines, and some perennial ornamentals, and it may cause significant economic losses in nurseries and plantations worldwide (Otten et al. 2008; Puławska 2010; Kuzmanović et al. 2018). Crown gall is caused by tumorigenic *Rhizobiaceae* strains, including multiple species from the genera *Agrobacterium*, *Allorhizobium*, and *Rhizobium*.

In general, members of the *Rhizobiaceae* family have diverse genome organization that may include circular chromosomes, linear and circular chromids, and multiple plasmids (Slater et al. 2009; Harrison et al. 2010; Ramírez-Bahena et al. 2014). The number and size of plasmids carried by *Rhizobiaceae* strains associated with crown gall may be highly variable (Albiach and Lopez 1992; Puławska et al. 1998). Plasmids are mobile genetic elements that play important roles in the ecology, adaptation and evolution of bacteria (Heuer et al. 2008; Heuer and Smalla 2012; Smalla et al. 2015). Accordingly, in *Rhizobiaceae* strains associated with crown gall disease, plasmids may carry pathogenetically and environmentally relevant traits associated with plant–bacterial interactions (Pappas and Cevallos 2011; Schierstaedt et al. 2019).

Tumor-inducing (Ti) plasmids are indispensable for the pathogenicity of agrobacteria. They are diverse replicons that show a mosaic structure composed of conserved and highly variable regions (Otten et al. 2008). Although Ti plasmids are generally well-studied replicons, complete high-quality and thoroughly annotated sequences are available for a limited number of Ti plasmids, including pTiC58 and pTiS4 (Slater et al. 2009), pTi-SAKURA (Suzuki et al. 2000), pTi-Octopine (Zhu et al. 2000), and pTiChry5 (Shao et al. 2018).

Ti plasmids are composed of the following elements: Transferred DNA (T-DNA), a virulence (*vir*) region, opine catabolism genes, a replication (*rep*) region, conjugative transfer genes (*tra* and *trb* loci), and uncharacterized and cryptic accessory regions. Conjugal transfer of Ti plasmids is regulated by a quorum-sensing (QS) mechanism and is induced by opines that are produced in crown gall tumors (Dessaux et al. 1998; Farrand 1998; White and Winans 2007; Faure and Lang 2014). Conjugal transfer genes are organized into *tra* and *trb* operons, which are Dtr (DNA transfer and replication; or MOB) and Mpf (mating pair formation) components, respectively. On the other hand, the T-DNA and *vir* region are essential for tumorigenesis in host plants. During an infection of the host plant by the tumorigenic *Rhizobiaceae* strain, T-DNA is transferred and integrated into the plant host genome through the process mainly mediated by *vir* genes (Gelvin 2017). VirB and VirD4 proteins form a second T4SS encoded on the Ti plasmid in addition to the Tra/Trb conjugal transfer system. Integrated T-DNA genes are not only associated with tumor-induction (oncogenes; Britton et al. 2008), but T-DNA also carries genes encoding biosynthesis of opines mentioned above. Opines are specific organic compounds that are mainly used as selective nutrient sources by a pathogen (Dessaux et al. 1998; Dessaux and Faure 2018a).

Different approaches have been used for the classification of Ti plasmids. Historically, classification of Ti plasmids was principally based on opine markers. Furthermore, based on incompatibility (Inc) properties, Ti plasmids and related root-inducing (Ri) plasmids have been classified into four Inc groups (Otten et al. 2008). In terms of their mobilization (MOB) systems, Ti plasmids described so far belong to the MOB_Q_ and MOB_P_ families, as determined by analysis of their TraA and VirD2 relaxase protein sequences, respectively (Christie and Gordon 2014). On the basis of TrbE and VirB4 MPF protein sequences, both T4SSs of Ti plasmids were classified into the MPF_T_ group.

Nonpathogenic and pathogenic *Rhizobiaceae* strains associated with crown gall disease may also harbor other ecologically important plasmids. Particularly interesting are so-called opine-catabolic (OC) plasmids. OC plasmids are specific group of plasmids that, like Ti plasmids, carry genes encoding the uptake and catabolism of opines, thus enabling their host bacteria to persist in tumor tissue. However, unlike Ti plasmids, OC plasmids do not contain *vir* genes and T-DNA required for pathogenicity. OC plasmids are found both in nonpathogenic and pathogenic *Rhizobiaceae* stains isolated from tumors or soil around diseased plants (Merlo and Nester 1977; Petit et al. 1983; Wabiko et al. 1990; Szegedi et al. 1999; Wetzel et al. 2014). Plasmids pAtK84b and pAoF64/95 carried by nonpathogenic *Rhizobium rhizogenes* (a.k.a. *Agrobacterium* biovar 2) strains K84 and F64/95, respectively, are the only OC plasmids that have been studied in more detail (Clare et al. 1990; Wetzel et al. 2014). Unlike tumorigenic strains and their Ti plasmids that were historically predominantly in research focus, data regarding the distribution, diversity, evolution and ecology of OC plasmids associated with crown gall tumors are still limited. Moreover, little is known about evolutionary relationships between Ti and OC plasmids.

Despite the significant volume of research, our understanding of the diversity and evolution of plasmids in pathogenic and nonpathogenic *Rhizobiaceae* strains associated with crown gall tumors is still in its infancy. In this study, Ti and OC plasmids in two tumorigenic and one nonpathogenic *R. rhizogenes* strains isolated from the same crown gall tumor on “Colt” cherry rootstock were sequenced and comparative genomic analyses were conducted. Consequently, we proposed a thorough scheme for classifying Ti and OC plasmids and investigated their evolutionary history.

## Materials and Methods

### Bacterial Strains

Two tumorigenic (C5.7 and C6.5) and one nonpathogenic (Colt5.8, also reported as C5.8, Puławska et al. 2016) strains were used in this study. All three strains were isolated from the same crown gall tumor on “Colt” cherry rootstock in Poland in 2008. They were previously identified as *R. rhizogenes*, while their tumor-inducing (Ti) ability was tested by PCR analysis and a pathogenicity assay on sunflower seedlings (Puławska et al. 2016). For PCR analysis targeting *noxA* gene involved in catabolism of nopaline (see below), we used collection of 93 nontumorigenic *Rhizobiaceae* strains isolated from crown gall tumors on various plants (supplementary table S1, Supplementary Material online).

### DNA Extraction

For Illumina MiSeq sequencing, plasmid DNA was extracted from bacterial cultures grown on Circlegrow (MP Biomedicals, France) for 24 h at 26 ˚C using the NucleoBond Xtra Midi kit (Macherey-Nagel, Duren, Germany) according to the manufacturer’s instructions. For PCR analysis, total genomic DNA of the strains was extracted from bacteria grown on King’s medium B (King et al. 1954) at 26 ˚C for 24 h according to the procedure described by Aljanabi and Martinez (1997).

### Plasmid Sequencing, Sequence Assembly, and Gap Closure

DNA sequencing was conducted using a whole-genome shotgun strategy on the Illumina MiSeq platform at Genomed S.A. (Warsaw, Poland). Paired-end libraries (insert size 300–500 bp) and mate-pair libraries (insert sizes 1.5–3 and 6–9 kb) were sequenced with 2 × 250 bp paired-end reads. Sequence processing and de novo assembly were performed using the CLC Genomics Workbench ver. 8.0.2 and A5-miseq pipeline ver. 20150522 (Coil et al. 2015).

Contigs with high coverage were considered to correspond to plasmid DNA. Moreover, BLAST searches (Johnson et al. 2008) were carried out to assign contigs to plasmids. PCR and standard Sanger sequencing were used for contig joining and gap closing. The obtained scaffolds were checked by mapping reads in CLC Genomics Workbench ver. 8.0.2 and visually inspected to correct misassemblies.

### Annotation

The IGS Annotation Engine was used for structural and functional annotation of the sequences (http://ae.igs.umaryland.edu/cgi/index.cgi; Last accessed on March, 2019. Galens et al. 2011). Manatee was used to view annotations (http://manatee.sourceforge.net/; Last accessed on March, 2019). Additionally, the plasmid sequences were automatically annotated by the Rapid Annotation Using Subsystem Technology (RAST; Aziz et al. 2008). Moreover, some loci were functionally annotated by blastp comparison against the NCBI non-redundant (nr) protein database (https://blast.ncbi.nlm.nih.gov/Blast.cgi; Last accessed on March, 2019), as well as by BLAST search tool of the KEGG database (Kanehisa et al. 2016). Insertion sequence (IS) elements were identified using the ISfinder tool (https://isfinder.biotoul.fr/; Last accessed on March, 2019. Siguier et al. 2006). Annotations were manually curated and submitted to NCBI GenBank (see below). The plasmid sequences were set to start with the predicted replication genes.

### Phylogenetic Analyses

Protein sequence alignments were conducted using MAFFT version 7 (Katoh et al. 2017). Unreliably aligned regions were identified and removed using Guidance2 (Sela et al. 2015). Columns with a confidence score below 0.93 were trimmed from the final alignment. Maximum likelihood (ML) trees were generated with PhyML 3.0 (Guindon et al. 2010) using aLRT SH-like method for branch support. The most suitable substitution models were determined by the Smart Model Selection (SMS) tool (Lefort et al. 2017) according to the Akaike information criterion (AIC) (Akaike 1974).

### Comparative Genomic Analyses

Whole plasmid sequence comparisons were performed using the BRIG (BLAST Ring Image Generator) programme ver. 0.95 (Alikhan et al. 2011). BRIG analysis involved the calculation of percentage of sequence identity and sequence coverage, in respect to the reference plasmid. The analysis was performed using BlastN option. Furthermore, plasmid sequences were compared by calculating average nucleotide identity (ANI) values using the JSpecies Web Service and ANIm algorithm (Richter et al. 2016). For comparison of smaller stretches of DNA, we employed the ANI Calculator, which uses the OrthoANIu algorithm (https://www.ezbiocloud.net/tools/ani; Last accessed on March, 2019. Yoon et al. 2017). Pairwise nucleotide identities for individual genes were calculated using the p-distance model with MEGA 7.0.21 software package (Kumar et al. 2016). NCBI BlastN and blastp (https://blast.ncbi.nlm.nih.gov/Blast.cgi; Last accessed on March, 2019) were also used for sequence comparisons at the nucleotide and amino acid levels, respectively.

### PCR Detection of *noxA* Gene Involved in Catabolism of Nopaline

Specific primers noxA-F (5′-GGTCTCATCAGCCGTTCGCT-3′) and noxA-R (5′-GACAGCAGCTTCCCTCAACACG-3′) targeting the *noxA* gene encoding D-nopaline dehydrogenase were designed in this study. The *noxA* gene is a part of the operon involved in the catabolism of nopaline and is carried by Ti and OC plasmids pTiC58 (Acc. No. AE007871), pTi-SAKURA (AB016260), pAtK84b (CP000630), and pF5.1b (CM004503). This primer set amplifies a 624 bp fragment of the *noxA* gene. The specificity of the primers was checked using the Primer-BLAST tool (https://www.ncbi.nlm.nih.gov/tools/primer-blast/; Last accessed on December, 2018) by searching the NCBI nr database (bacteria), as well as by PCR analysis using reference agrobacteria. In this study, we used primers noxA-F/noxA-R for the detection of the *noxA* gene in a collection of nontumorigenic *Rhizobiaceae* strains isolated from crown gall tumors on various plants (supplementary table S1, Supplementary Material online).

The PCR amplifications were performed in a 15 μl mixture containing 1× DreamTaq Green Buffer (includes 20 mM MgCl_2_; Thermo Scientific, Vilnius, Lithuania), 200 μM of each dNTP, 0.5 μM of each primer, 0.3 U of DreamTaq DNA polymerase (Thermo Scientific, Vilnius, Lithuania), and 1 μl of template DNA. The thermal profile was as follows: Initial denaturation at 94 ˚C for 5 min, 35 cycles of denaturation at 94 ˚C for 30 s, annealing at 62 ˚C for 30 s, elongation at 72 ˚C for 45 s, and final extension at 72 ˚C for 5 min.

### Nucleotide Sequence Accession Numbers

The whole plasmid sequences obtained in this study have been deposited at GenBank (NCBI) under the following accession numbers: MF511177 (pTiC5.7), MK318973 (pOC-Colt5.8), and MK318986 (pTiC6.5).

## Results

### General Characteristics of Ti and OC Plasmids

Strains C5.7 and C6.5 harbored Ti plasmids designated pTiC5.7 and pTiC6.5, respectively, which is consistent with their ability to induce tumors (Puławska et al. 2016). On the other hand, strain Colt5.8 carried the OC plasmid designated pOC-Colt5.8.

All sequenced plasmids were circular. The size of sequenced Ti (pTiC5.7/pTiC6.5) and OC (pOC-Colt5.8) plasmids was 218,413 bp and 172,401 bp, respectively (table 1). Their GC content was 56.8% (pTiC5.7/pTiC6.5) and 59.2% (pOC-Colt5.8). Plasmids pTiC5.7/pTiC6.5 and pOC-Colt5.8 contained 213 and 181 ORFs (table 1).

**Table 1:** Characteristics of Ti and OC Plasmids of Three *Rhizobium rhizogenes* Strains

Plasmid	pTiC5.7^a^	pOC-Colt5.8	pTiC6.5^a^
Acc. no.	MF511177	MK318973	MK318986
Host strain	*R. rhizogenes* C5.7	*R. rhizogenes* Colt5.8	*R. rhizogenes* C6.5
Type	Tumor-inducing	Opine-catabolic	Tumor-inducing
Functions	Virulence; nopaline and agrocinopine catabolism	Nopaline and agrocinopine catabolism	Virulence; nopaline and agrocinopine catabolism
Size (bp)	218,413	172,401	218,413
GC content (%)	56.8	59.2	56.8
ORFs^b^	213	181	213
REP type^c^	*repABC*	*repABC*	*repABC*
MOB family^d^	MOB_Q2_ (*tra*);	MOB_Q2_	MOB_Q2_ (*tra*);
	MOB_P2_ (*vir*)		MOB_P2_ (*vir*)
T4SS type^e^	MPF_T_ (*trb*);	MPF_T_	MPF_T_ (*trb*);
	MPF_T_ (*vir*)		MPF_T_ (*vir*)

a Ti plasmids analyzed carried two independent sets of MOB and T4SS genes.

b Open reading frames.

c Type of replication-partitioning system.

d Mobilization families determined by phylogenetic analysis of conjugative relaxase proteins.

e Type IV secretion system groups determined by phylogenetic analysis of TrbE and VirB4 mating pair formation (MPF) proteins.

### Genetic Maps of Ti and OC Plasmids

**Fig. 1.**
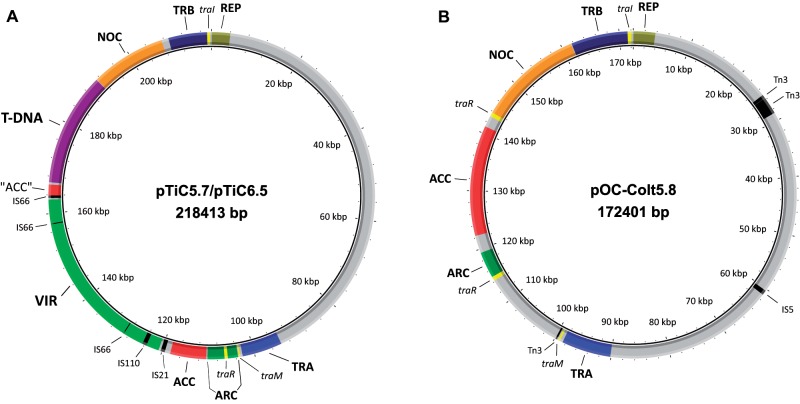
—Genetic maps of pTiC5.7/pTiC6.5 (left) and pOC-Colt5.8 (right). The genetic regions and specific genes are indicated with respect to their putative functions as follows: REP, replication-partitioning system; TRA, DNA transfer and replication (Dtr) region; TRB, mating pair formation (Mpf) region; VIR, virulence region; T-DNA, transferred DNA; ACC, transport and catabolism of agrocinopine region; ARC, agrocinopine regulation of conjugation operon, of which regulatory gene *traR* is a member; NOC, transport and catabolism of nopaline and nopalinic acid (additional *traR* gene is a part of this region in pOC-Colt5.8); genes *traM* and *traI* involved in regulation of conjugative transfer are also shown. Transposable elements are indicated in black and additionally listed in supplementary table S2, Supplementary Material online. In pTiC5.7/pTiC6.5, a DNA fragment between VIR and T-DNA regions (“ACC”) that exhibited high degree of nucleotide identity to several genes contained in the ACC region of OC plasmids pOC-Colt5.8 and pAtK84b is shown. The figure was generated using BRIG.

Plasmids pTiC5.7/pTiC6.5 and pOC-Colt5.8 carried genetic modules associated with replication-partitioning (REP) and conjugal transfer (TRA and TRB regions) (fig. 1). They also contained regulatory genes *traR*, *traI*, and *traM* involved in the QS regulatory system (fig. 1). In addition to conjugal transfer genes, pTiC5.7/pTiC6.5 carried genes for a second DNA transfer system associated with virulence (VIR region; fig. 1*A*). Moreover, these plasmids carried T-DNA, which is also required for pathogenicity, as well as regions involved in uptake and catabolism of nopaline and nopalinic acid (NOC region) and agrocinopine (ACC region), and a region for agrocinopine regulation of conjugation (ARC region). Distantly from the agrocinopine region, between the VIR and T-DNA regions, plasmids pTiC5.7/pTiC6.5 contain a region (“ACC”) showing a high degree of nucleotide identity to several genes contained in the ACC region of OC plasmids pOC-Colt5.8 and pAtK84b (fig. 1*A*). Plasmid pOC-Colt5.8 carried ACC, ARC, and NOC regions, as Ti plasmids, but lacked VIR and T-DNA regions required for pathogenicity (fig. 1*B*).

**Fig. 2.**
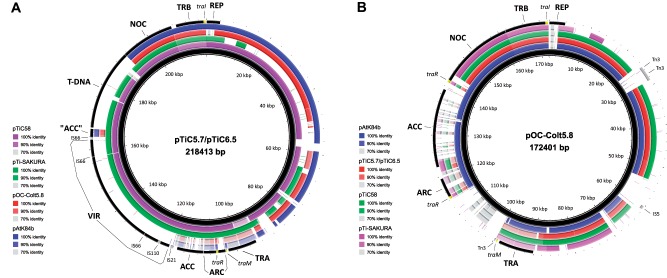
—Comparison of pTiC5.7/pTiC6.5 (*A*) and pOC-Colt5.8 (*B*), with related plasmids pTiC58 (Acc. No. AE007871), pTi-SAKURA (AB016260), and pAtK84b (CP000630). The innermost ring (colored black) and the coordinates (indicated in kilobases) represent pTiC5.7/pTiC6.5 (*A*) and pOC-Colt5.8 (*B*). The colored rings (from inner to outer ring) portray related plasmid sequences, as indicated in figure legend. Genes and regions of interest from pTiC5.7/pTiC6.5 (*A*) and pOC-Colt5.8 (*B*) are indicated on the outermost ring and are labeled as in figure 1. In pTiC5.7/pTiC6.5, a DNA fragment between VIR and T-DNA regions (“ACC”) that exhibited high degree of nucleotide identity to several genes contained in the ACC region of OC plasmids pOC-Colt5.8 and pAtK84b is shown. The figure was generated using BRIG.

Various transposable elements (TE) were identified in plasmids pTiC5.7/pTiC6.5 and pOC-Colt5.8 (figs. 1 and 2; supplementary table S2, Supplementary Material online). We identified TE elements belonging to families IS5, IS21, IS66, IS110, and Tn3 (figs. 1 and2; supplementary table S2, Supplementary Material online). Overall, plasmids pTiC5.7/pTiC6.5 and pOC-Colt5.8 carried distinct sets of TE (figs. 1 and2; supplementary table S2, Supplementary Material online).

On the basis of genes and gene clusters responsible for the synthesis and catabolism of opines, pTiC5.7 and pTiC6.5 were designated as nopaline-type Ti plasmids. Since pOC-Colt5.8 also possessed a similar genetic region for the catabolism of nopaline, we designated this plasmid as a nopaline-type OC plasmid (table 2).

**Table 2:** Characterization of Backbone and Accessory Regions of pTiC5.7/pTiC6.5 and pOC-Colt5.8

	pTiC5.7/pTiC6.5^a^	pOC-Colt5.8^a^
Opine-type	Nopaline-type Ti plasmid	Nopaline-type OC plasmid
Opine synthesis genes	Nopaline and nopalinic acid (*nos*), agrocinopines A+B (*acs*)	—
Opine catabolism genes	Nopaline and nopalinic acid (NOC), agrocinopines A+B (ACC)	Nopaline and nopalinic acid (NOC), agrocinopines A+B (ACC)
*repABC*	1 locus	1 locus
	pAtK84b-like (100% ANI)	Similar to pAtS4c (88.4% ANI)
TRA	pTi-SAKURA-like (98% ANI)	Similar to pAtK84b (87.26% ANI)
TRB	pAtK84b-like (100% ANI)	Similar to pAtK84b (92.28% ANI)
T-DNA composition^b^	*a*, *b*, *c*, *c′*, *d*, *e*, *f*, *acs*, *5*, *iaaH*, *iaaM*, *ipt*, *6b*, *3′*, and *nos*	—
VIR	pTiC58/pTi-SAKURA-like (>99% ANI)	—
NOC	pAtK84b-like^c^ (99.35% ANI)	pAtK84b-like (97.67% ANI)
ACC/ARC	pTiC58/pTi-SAKURA-like (>99% ANI)	pAtK84b-like (96.68% ANI)
QS regulatory genes^d^	*traR*	*traR* (NOC) and *traR* (ARC)
	*traI* (pAtK84b-like, 100% ANI)	*traI* (pAtK84b-like, 99.06% ANI)
	*traM* (pTiC58/pTi-SAKURA-like, >98% ANI)	*traM* (similar to pAtK84b, 92.88% ANI)

a Classification of particular genetic regions was based on sequence comparison by average nucleotide identity (ANI) calculations. ANI values are indicated in parentheses. Fractions of the sequences that were aligned and included into the ANI calculations (sequence coverages) were >98%, except for comparison of ACC/ARC region of plasmids pOC-Colt5.8 and pAtK84b (sequence coverage >96%).

b Considering distinct organization of pTiC5.7/pTiC6.5 T-DNA (in respect to plasmids whose sequences are available), we did not conduct ANI calculations.

c Although NOC region of pTiC5.7/pTiC6.5 was highly similar to corresponding region of pAtK84b, the latter one carried *traR* gene linked to the NOC region.

d Regulatory gene *traR* was included into sequence comparison as a constitutive part of NOC and/or ACC/ARC regions.

### Whole-Sequence Based Comparisons of Plasmids

The nucleotide sequences of pTiC5.7 and pTiC6.5 were almost identical, with only three single nucleotide polymorphisms (SNPs). Two neighboring SNPs were located within the gene annotated to encode Toprim-like family protein (locus_tag pTiC5.7/pTiC6.5_68) and resulted in one amino acid change. The third SNP was located in the intergenic region between the *tzs* and *virA* genes.

Ti plasmids pTiC5.7/pTiC6.5 (218,413 bp in size) showed 96.97% ANI with pTiC58 (214,233 bp in size), which was based on >93% of the aligned sequence for both plasmids. Indeed, whole plasmid sequence comparison indicated that most of the regions between these two plasmids were conserved (fig. 2*A*). Although pTiC5.7/pTiC6.5 showed high ANI (>95%) with pTi-SAKURA, it was based on ∼70% of aligned sequences, as evident by whole plasmid sequence comparison of these plasmids (fig. 2*A*). Accordingly, pTiC5.7/pTiC6.5 shared a large portion of accessory genes located between the REP and TRA regions with pTiC58 but not with pTi-SAKURA (fig. 2*A*). Interestingly, this accessory region was also conserved in pOC-Colt5.8 and even more so in pAtK84b (fig. 2*A*). Plasmid pOC-Colt5.8 (172,401 bp in size) was most closely related to pAtK84b (184,668 bp in size), with which it exhibited 95.66% ANI, based on 78.99% and 73.77% of the aligned sequences of pOC-Colt5.8 and pAtK84b, respectively.

### Replication and Partitioning Systems

**Fig. 3.**
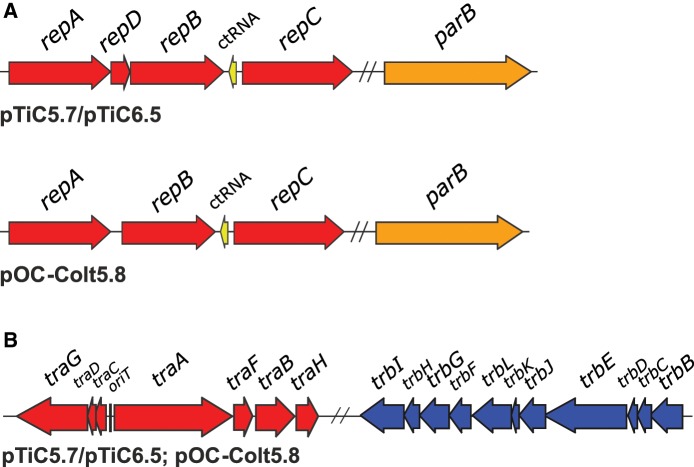
—Genetic organization of putative replication/partitioning (*A*) and mobilization/conjugation (*B*) modules of plasmids sequenced in this study. Putative replication/partitioning genes are colored in red. Additional orphan *parB* genes are colored in orange. Genes for nontranslated counter-transcribed RNA (ctRNA) are colored in yellow. Putative mobilization (Dtr or MOB) genes are colored in red, whereas putative mating-pair formation (Mpf) genes are colored in blue. Putative origin of transfer (*oriT*) is indicated by vertical line. The double slash indicates that genetic regions are located separately.

Plasmids pTiC5.7/pTiC6.5 and pOC-Colt5.8 possessed the *repABC* cassette, composed of *repA*, *repB*, and *repC* genes (table 1, fig. 3*A*). For Ti plasmids studied, we also identified putative *repD* gene, between *repA* and *repB* genes (fig. 3*A*). Although the role of *repD* in plasmid biology is still unclear, it was hypothesized that this gene might ensure the expression of *repB* and *repC* (Chai and Winans 2005). *repD* gene of pTiC5.7/pTiC6.5 comprised two RepB-binding *par* sites required for plasmid partitioning, that were identical as those found in pTiC58 and pTiR10 (Chai and Winans 2005; data not shown). However, the whole nucleotide sequence of *repD* gene of pTiC5.7/pTiC6.5 showed only 75.2% and 56.4% of identity with corresponding gene of plasmids pTiC58 and pTiR10, respectively. Plasmids pTiC5.7/pTiC6.5 and pOC-Colt5.8 carried an additional plasmid-partitioning gene *parB* annotated to encode ParB family partition protein (RepB homologue). Putative *parB* gene was separated from the *repABC* cassette (fig. 3*A*), which was also the case for pTiC58 (Atu6103) and pAtK84b (Arad_14050). In all *repABC* cassettes, a gene for nontranslated counter-transcribed RNA (ctRNA) was identified *in silico* between *repB* and *repC* genes (fig. 3*A*).

We performed phylogenetic analysis by using individual RepA, RepB, and RepC protein sequences representing respective genes from the *repABC* cassette of the plasmids studied. We also included corresponding sequences from related *Rhizobiaceae* plasmids into phylogenetic analysis. Generally, the *repABC* proteins of the studied plasmids were phylogenetically related to those located in plasmids of various *Rhizobiaceae* strains (supplementary fig. S1, Supplementary Material online).

With the exception of the pOC-Colt5.8, the *repABC* region was also more or less conserved (ANI >94%) between all plasmids studied (fig. 2). Interestingly, pTiC5.7/pTiC6.5 and pAtK84b have identical nucleotide sequences corresponding to the *repAB*C region (table 2), suggesting that these plasmids most likely belong to the same Inc group. On the basis of sequence comparison, the *repABC* region of pOC-Colt5.8 was designated as similar to the plasmid pAtS4c carried by *Allorhizobium vitis* strain S4 (table 2). As suggested by phylogenetic analysis, pOC-Colt5.8 was more distantly related based on *repA* and *repB* gene phylogeny (supplementary figs. S1*A* and S1*B*, Supplementary Material online), although it was related to plasmids pTiC5.7/pTiC6.5, pAtK84b, pTiC58 and pTi-SAKURA based on *repC* gene phylogeny (supplementary fig. S1*C*, Supplementary Material online).

### Mobilization and Conjugation Systems

Plasmids pTiC5.7/pTiC6.5 and pOC-Colt5.8 carried a complete set of putative transfer genes (Dtr + Mpf), suggesting that they are conjugative plasmids. In particular, they carried all of the genes required for conjugative transfer (TRA and TRB regions; fig. 3*B*), including QS regulatory genes (fig. 1), resembling gene content and organization to those of other Ti (e.g., pTiC58) and OC (e.g., pAtK84b) plasmids. Between the *traC* and *traA* genes, we detected *in silico* a probable origin of transfer (*oriT*) region of pTiC5.7/pTiC6.5 and pOC-Colt5.8, which was strongly conserved with the *oriT* region of pTiC58 that was previously characterized (supplementary fig. S2, Supplementary Material online) (Cook and Farrand 1992).

Genes encoding conjugative relaxases (*traA*/*virD2*), and T4SS proteins VirB4/TrbE are considered core genes of conjugation and are proposed as a phylogenetic markers for classification of plasmids, or their transfer systems (Garcillan-Barcia et al. 2009; Smillie et al. 2010). Therefore, we performed phylogenetic analysis to classify transfer systems of plasmids sequenced in this study. Plasmids pTiC5.7/pTiC6.5 and pOC-Colt5.8 clustered within a MOB_Q2_ group comprising plasmids with a type I conjugation system regulated by a QS mechanism (Ding and Hynes 2009; Ding et al. 2013), as observed in a TraA phylogenetic tree that included MOB_Q2_ representatives (supplementary fig. S3*A*, Supplementary Material online). We also analyzed the corresponding protein sequences of a second DNA transfer system (*vir*) of pTiC5.7/pTiC6.5 involved in T-DNA transport to plant cells. Their transfer systems were classified into the MOB family MOB_P2_ based on the VirD2 relaxase protein sequence (supplementary fig. S3*B*, Supplementary Material online). Phylogenetic analysis based on TrbE/VirB4 MPF protein sequence (supplementary fig. S4, Supplementary Material online; table 1) suggested that T4SSs (*trb* and *vir*) of plasmids sequenced were classified into the MPF_T_ group.

Regions TRA and TRB involved in plasmid conjugative transfer were more or less conserved between all Ti and OC plasmids used for comparison (fig. 2). To classify these plasmid regions, we compared complete TRA (*traG*-*traH*) and TRB (*trbI*-*trbB*) regions among the plasmids studied. Considering the high ANI values obtained, we classified the TRA and TRB regions of pTiC5.7/pTiC6.5 as pTi-SAKURA-like and pAtK84b-like, respectively (table 2). Interestingly, TRB regions of pTiC5.7/pTiC6.5 and pAtK84b were identical. Furthermore, ANI calculation and whole plasmid sequence comparison (fig. 2*B*) suggested lower degree of nucleotide identity of the TRA and TRB regions of pOC-Colt5.8, comparing to other plasmids included into comparison. Because TRA and TRB regions of pOC-Colt5.8 showed some ANI (∼87–92%) to corresponding regions of pAtK84b, they were designated as similar to pAtK84b (table 2). On the basis of nucleotide identity, *traI* genes of plasmid sequenced in this study were classified as pAtK84b-like (>99%). Regulatory gene *traM* of plasmids pTiC5.7/pTiC6.5 and pOC-Colt5.8 was classified as pTiC58/pTi-SAKURA-like and as similar to pAtK84b, respectively (table 2).

Notably, whole plasmid sequence comparison (fig. 2*A*) suggested weak nucleotide identity between TRA regions of pTiC5.7/pTiC6.5 (including pTi-SAKURA) and pTiC58 in a stretch containing *traG*, *traD*, *traC*, and *traA* genes. Therefore, we reconstructed phylogenies based on each gene of the TRA region and included related Ti and OC plasmids. Indeed, in TraG, TraD, TraC, and TraA phylogenetic trees, pTiC58 was more closely related to other Ti and OC plasmids than to the Ti plasmids pTiC5.7/pTiC6.5 and pTi-SAKURA, unlike TraF, TraB, and TraH phylogenies (supplementary fig. S5, Supplementary Material online). Plasmid pOC-Colt5.8 was clustered separately from other plasmids, although it was closely related to pAtK84b in TraG phylogenetic tree (supplementary fig. S5, Supplementary Material online). Furthermore, as observed on phylogenetic trees based on individual genes of the TRB region, pOC-Colt5.8 was clustered either with plasmids pTiC5.7/pTiC6.5 or separately from other plasmids included into analysis (supplementary fig. S6, Supplementary Material online). We also reconstructed phylogenies based on QS regulatory genes *traM* and *traI*. In TraM phylogenetic tree, pTiC5.7/pTiC6.5 clustered with pTiC58 and pTi-SAKURA, whereas the pOC-Colt5.8 formed an independent branch (supplementary fig. S7*A*, Supplementary Material online). On the other hand, in TraI phylogenetic tree, plasmids pTiC5.7/pTiC6.5, pOC-Colt5.8, and pAtK84b clustered closely together, whereas plasmids pTiC58 and pTi-SAKURA formed a separate branch (supplementary fig. S7*B*, Supplementary Material online). Taken together, phylogenetic trees based on single genes of either TRA or TRB regions, including QS regulatory genes *traM* and *traI*, generally showed different topologies (supplementary figs. S5–S7, Supplementary Material online).

### Accessory Regions of pTiC5.7/pTiC6.5 and pOC-Colt5.8

#### T-DNA

**Fig. 4.**
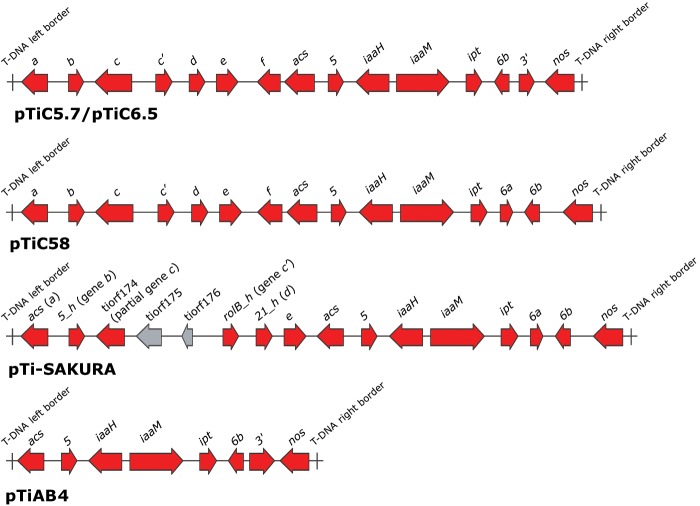
—Genetic organization of the T-DNA of pTiC5.7/pTiC6.5 and comparison to related Ti plasmids. Putative T-DNA genes are colored in red. Insertion elements and transposons are colored in gray. T-DNA border sequences are indicated by vertical lines.

The T-DNA region in pTiC5.7/pTiC6.5 was located between the VIR and NOC regions in a stretch of ∼24 kbp. We identified putative T-DNA genes and followed gene nomenclature as that used in the paper of Otten et al. (1999). The T-DNA of pTiC5.7/pTiC6.5 was composed of putative genes arranged in the following order: *a*, *b*, *c*, *c′*, *d*, *e*, *f*, *acs*, *5*, *iaaH*, *iaaM*, *ipt*, *6b*, *3′*, and *nos* (fig. 4). We compared the gene content of T-DNA of pTiC5.7/pTiC6.5 with T-DNAs of other nopaline-type Ti plasmids characterized so far, including plasmids pTiC58 (Otten et al. 1999), pTi-SAKURA (Suzuki et al. 2000) and nopaline-type Ti plasmid carried by *All. vitis* AB4 (Otten and De Ruffray 1994).

Generally, the T-DNA organization and gene content of pTiC5.7/pTiC6.5, pTiC58, and pTi-SAKURA are similar (fig. 4). However, unlike pTiC58 and pTi-SAKURA, the T-DNA of pTiC5.7/pTiC6.5 carried gene *3′*, whereas the gene *6a* was absent. More specifically, genes located on the left part of the T-DNA (genes *a*-to-*acs*) exhibited high nucleotide identity (>99%) with homologous loci harbored by pTiC58 and/or pTi-SAKURA. The remaining gene *5*, located between the left and right part of the T-DNA of pTiC5.7/pTiC6.5, showed a relatively lower degree of nucleotide identity (∼91–92%) with all three Ti plasmids (pTiC58, pTi-SAKURA and pTiAB4) that were used for comparison. Interestingly, the left part of gene *5* (coordinates 1–267) exhibited high nucleotide identity (97.4%) to the corresponding gene fragment of pTiC58, whereas the right part (coordinates 268–684) showed high nucleotide identity (97.6%) to the corresponding gene region of pTiAB4.

On the other hand, genes located on the right part of the T-DNA of pTiC5.7/pTiC6.5 (*iaaH*-to-*nos*) showed a high degree of nucleotide identity (∼95–99%) to homologous T-DNA genes in pTiAB4 and were more distantly related to those carried by pTiC58 and pTi-SAKURA (<94% nucleotide identity). Notably, genes *6b* and *nos* of pTiC5.7/pTiC6.5 shared just ∼62% and ∼88% nucleotide identity, respectively, with corresponding genes of plasmids pTiC58 and pTi-SAKURA. Interestingly, the 4.7 kb right part of the T-DNA (*ipt*-*6b*-*3′*-*nos*) of pTi82.139 (Acc. No. X74123) was almost identical (only eight SNPs) to corresponding region of pTiC5.7/pTiC6.5. However, we could not extend comparison to the left part of the T-DNA, because only its right part was available for pTi82.139 (Drevet et al. 1994). Nonetheless, as indicated above for pTiC5.7/pTiC6.5, it has also been suggested that the left part of the T-DNA of pTi82.139 and pTiC58 are highly conserved (Michel et al. 1990; Drevet et al. 1994). pTi82.139 is a nopaline-type Ti plasmid carried by *R. rhizogenes* strain 82.139 isolated from *Prunus avium* (Michel et al. 1990).

#### VIR Region

**Fig. 5.**
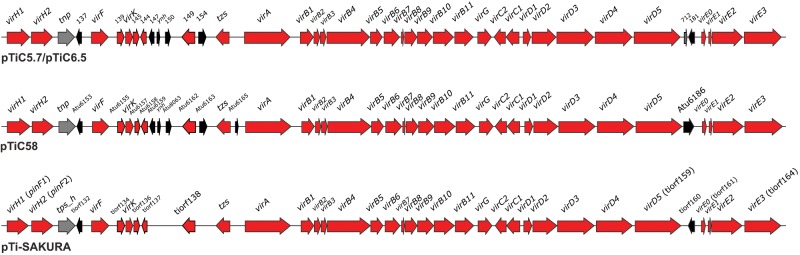
—Genetic organization of VIR region of pTiC5.7/pTiC6.5 and comparison to other related Ti plasmids. Genes colored in red are members of the *vir* regulon. Insertion elements and transposons are colored in gray. All other genes are colored in black. For pTiC5.7/pTiC6.5, numbers above genes correspond to locus_tag numbers.

The VIR region of pTiC5.7/pTiC6.5 was located in a stretch of ∼42 kbp, downstream the ACC region (fig. 1*A*). The VIR region of pTiC5.7/pTiC6.5 was highly similar in gene content and organization to the VIR regions of nopaline-type Ti plasmids pTiC58 and pTi-SAKURA (fig. 5) (Rogowsky et al. 1990; Cho and Winans 2005). On the other hand, the VIR region of so far fully sequenced and characterized octopine- (pTi-Octopine; Zhu et al. 2000), chrysopine- (pTiChry5; Shao et al. 2018), agropine- (pTiBo542; Oger et al. unpublished data), and vitopine-type (pTiS4; Slater et al. 2009) Ti plasmids differed in gene content and organization, as well as in nucleotide sequence of common *vir* genes (data not shown).

As pTiC58 and pTi-SAKURA, pTiC5.7/pTiC6.5 carried *virB*, *virC*, *virD*, *virE*, and *virH* operons, including genes *tzs*, *virA*, *virF*, *virG*, and *virK* (fig. 5). It has been suggested that some additional genes are also members of the *vir* regulon in pTiC5.8, including *atu6155*, *atu6157*, *atu6158*, *atu6162*, and *atu6163R* (complementary to *atu6163*; Cho and Winans 2005). In this respect, we found homologs of genes *atu6155* (pTiC5.7/pTiC6.5_139), *atu6157* (pTiC5.7/pTiC6.5_145), *atu6158* (pTiC5.7/pTiC6.5_144), and *atu6162* (pTiC5.7/pTiC6.5_149) in pTiC5.7/pTiC6.5. Putative ORFs tiorf134, tiorf136, tiorf137, and tiorf138 in pTi-SAKURA also showed homology to *atu6155*, *atu6157*, *atu6158*, and *atu6162*, respectively, and might be a part of the *vir* regulon. However, the sequence of the gene *atu6163R* is unknown, and we could not check for its presence in pTiC5.7/pTiC6.5.

Whole plasmid sequence comparison (fig. 2*A*) suggested the high conservation of the VIR region among nopaline-type Ti plasmids. In particular, the VIR region of pTiC5.7/pTiC6.5, pTiC58 and pTi-SAKURA shared >99% ANI (table 2).

#### Gene Regions Associated with Opine Catabolism

Plasmids pTiC5.7/pTiC6.5 and pOC-Colt5.8 carried putative genes for utilization of nopaline, nopalinic acid, and agrocininopines A + B, as the classical nopaline-type Ti plasmid pTiC58 (Zanker et al. 1992; Kim and Farrand 1997) or nopaline-catabolic plasmid pAtK84b (Clare et al. 1990).

**Fig. 6.**
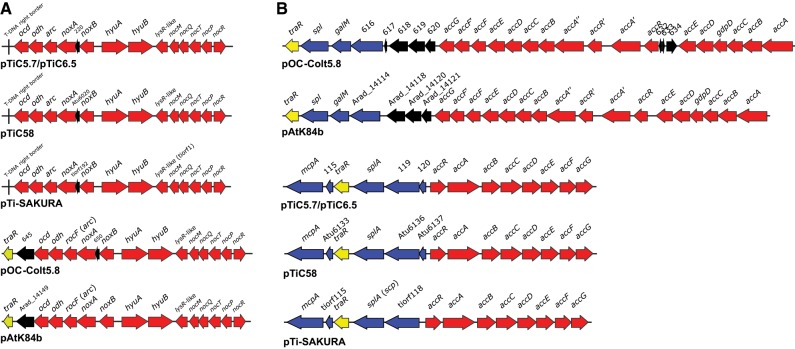
—Genetic organization of NOC (*A*) and ACC/ARC (*B*) regions of plasmids pTiC5.7/pTiC6.5 and pOC-Colt5.8, and comparison to other related Ti plasmids. Putative genes involved in nopaline, nopalinic acid and agrocinopines catabolism are colored in red. Genes colored in blue are members of the ARC operon. *traR* genes are colored in yellow. All other genes are colored in black. Vertical lines indicate T-DNA border sequences. For pTiC5.7/pTiC6.5 and pOC-Colt5.8, numbers above genes correspond to locus_tag numbers.

In pTiC5.7/pTiC6.5, the NOC region is located directly adjacent to the T-DNA (fig. 1*A*). This region lies between the ACC and TRB regions in pOC-Colt5.8 (fig. 1*B*). The NOC region was located in a stretch of ∼16 kbp and exhibited highly similar gene organization and conservation among pTiC5.7/pTiC6.5 and pOC-Colt5.8 and related Ti and OC plasmids included in the analysis (figs. 2 and 6*A*). However, unlike Ti plasmids, pOC-Colt5.8 carried the *traR* gene adjacent to the NOC region, as pAtK84b (figs. 1 and 6*A*). The NOC regions of pTiC5.7/pTiC6.5 and pOC-Colt5.8 showed the highest ANI to pAtK84b (table 2).

The ACC/ARC region of pTiC5.7/pTiC6.5 was located adjacent to the VIR region in a stretch of ∼15 kbp (fig. 1*A*). In pOC-Colt5.8, the ACC/ARC region was larger (∼27 kbp) and located in proximity to the NOC region (fig. 1*B*). The organization and gene content of the ACC/ARC region differed between pTiC5.7/pTiC6.5 and pOC-Colt5.8 and were highly similar to the related Ti (pTiC58 and pTi-SAKURA) and OC (pAtK84b) plasmids, respectively (fig. 6*B*). The ACC/ARC regions differed in organization and/or sequence in respect to other plasmids carrying ACC/ARC region, such pTiChry5 (Shao et al. 2018), pTiBo542 (Oger et al. unpublished data), and pAoF64/95 (Wetzel et al. 2014).

Taken together, ACC/ARC region was strongly conserved within nopaline-type Ti plasmids analyzed (fig. 2). The ACC/ARC region of pTiC5.7/pTiC6.5 exhibited very high ANI to pTiC58 and pTi-SAKURA (table 2). The ACC/ARC regions of pOC-Colt5.8 showed the highest ANI to the corresponding region of pAtK84b (table 2). As expected, considering the gene content (figs. 2 and 6*B*), weak nucleotide identity was observed between ACC/ARC regions of nopaline-type Ti and OC plasmids. For pTiC5.7/pTiC6.5 and pOC-Colt5.8, the ACC/ARC region shared only 75.70% ANI for <22% of sequence coverages.

Interestingly, plasmids pTiC5.7/pTiC6.5, including pTiC58 and pTi-SAKURA, carried a DNA fragment between VIR and T-DNA regions that exhibited a high degree of nucleotide identity to several genes contained in the ACC region of OC plasmids pOC-Colt5.8 and pAtK84b (figs. 1*A* and 2*A*). This DNA fragment contained ORFs that showed homology to genes *accF′*, *accG*, and *traR*, as well as to pOC-Colt5.8_619 and 620 (for pOC-Colt5.8), and Arad_14120 and 14121 (for pAtK84b). Sequence comparison suggested that all ORFs except pTiC5.7_190/pTiC6.5_190 might produce truncated and most likely nonfunctional proteins.

#### Additional Accessory Regions

Plasmids pTiC5.7/pTiC6.5 and pOC-Colt5.8 carried large ∼86–89 kb accessory region between REP and TRA regions (fig. 1), sharing ∼96% ANI based on ∼80% of sequence aligned. These plasmids had large portions of accessory region in common with pTiC58 and pAtK84b (fig. 2). On the other hand, pTi-SAKURA had a distinct accessory region showing low homology with pTiC5.7/pTiC6.5, and pOC-Colt5.8 (fig. 2).

The accessory region of pTiC5.7/pTiC6.5 and pOC-Colt5.8 comprised a number of putative ORFs encoding various enzymes, ABC transporters, and transcriptional regulators. For instance, they carried putative ABC transporter gene clusters that might be involved in the import of aliphatic sulfonates (*ssuABC*), peptides (*dfpABCD*), and ribose (*rbsABC*). Moreover, they carried a putative gene *aiiB* encoding putative N-acyl homoserine lactone (acyl-HSL) hydrolases. This gene was also present in pTiC58 and pAtK84b but not in pTi-SAKURA. The accessory region of pTiC5.7/pTiC6.5 and pOC-Colt5.8 carried numerous putative genes encoding hypothetical proteins.

Plasmid pOC-C58 carried another ∼15 kb accessory region between TRA and ARC regions (fig. 1). However, this region showed low homology to the other Ti and OC plasmids used for comparison. It contained genes encoding enzymes agmatinase/arginase, oxidoreductases (predominantly monooxygenases), oligopeptide ABC transporters and TetR/AcrR family transcriptional regulators. Interestingly, this region showed certain level of homology (79% nucleotide identity; Query cover: 40%) to the chromid of *R. rhizogenes* K84.

### Widespread Distribution of Nopaline-Catabolic Genes

By using newly designed primers, we tested the presence of *noxA* gene involved in catabolism of nopaline in various *Rhizobiaceae* strains that were negative in the pathogenicity assay and PCR using primers complementary to the *tms2* (*iaaH*) gene located within the T-DNA of the Ti plasmid (supplementary table S1, Supplementary Material online) (Puławska et al. 2016). Of the 93 strains tested, 75 produced positive amplification signals, including diverse *Agrobacterium* spp., *Pararhizobium* spp. and *Rhizobium* spp. isolated from tumors of different plant species (supplementary table S1, Supplementary Material online). The *noxA* gene seems to be exclusively plasmid-borne based on our mining of NCBI GenBank. Therefore, the results of PCR analysis suggested that the strains analyzed most likely harbor OC plasmids with genes involved in the catabolism of nopaline.

## Discussion

### Both Ti and OC Plasmids Were Associated with the Same Crown Gall Tumor

Ti and OC plasmids play a key role in the ecology and lifestyle of various *Rhizobiaceae* strains associated with crown gall disease. In this study, we sequenced Ti and OC plasmids in three *R. rhizogenes* strains originating from the same crown gall tumor and studied their diversity and evolution. In this respect, strains C5.7 and C6.5 carry almost identical Ti plasmids (pTiC5.7/pTiC6.5), whereas the strain Colt5.8 carries OC plasmid pOC-Colt5.8. Strains C5.7 and C6.5 are thus able to construct a specific opine-rich ecological niche in their host plants. Opines produced in tumors therefore serve as a selective nutrient source for tumorigenic bacterium and induce conjugative transfer of Ti plasmids (reviewed in Dessaux et al. 1998; Farrand 1998; Dessaux and Faure 2018a). On the other hand, our results suggested that the strain Colt5.8 is also able to catabolize opines and exploit crown gall tumors.

The GC content of Ti plasmids sequenced so far can vary from 54.5% (pTiChry5) to 56.7% (pTiC58 or pTiS4). Ti plasmids pTiC5.7/pTiC6.5 described here have a GC content of 56.8%, which was slightly above this range. The plasmid pOC-Colt5.8 had a higher GC content of 59.2%, which was similar to the OC plasmid pAtK84b (59%). The GC content of these OC plasmids was therefore closer to the GC content of total genomic DNA of the species *R. rhizogenes* (59.9%), as determined for the strain K84 (Slater et al. 2009). Its chromosome and chromid have GC contents of 60.3% and 59.8%, respectively.

As Ti and OC plasmids described so far, pTiC5.7/pTiC6.5 and pOC-Colt5.8 belonged to the *repABC* family of plasmids. They carried a complete set of putative transfer genes (TRA and TRB regions) with type I conjugation system regulated by a QS mechanism (Ding and Hynes 2009; Ding et al. 2013). Ti and OC plasmids sequenced in this study contained putative gene clusters responsible for the catabolism of opines nopaline and agrocinopine. In addition to the region involved in catabolism of agrocinopine, plasmids pTiC5.7/pTiC6.5 and pOC-Colt5.8 contained a putative region ARC. Therefore, agrocinopine likely serves as the conjugal opine and induces transfer of pTiC5.7/pTiC6.5 and pOC-Colt5.8, as previously shown for pTiC58 and pAtK84b (Ellis et al. 1982; Piper et al. 1999; Oger and Farrand 2002). Unlike pTiC5.7/pTiC6.5, pOC-Colt5.8 contained a second copy of the *traR* gene within the nopaline catabolic (NOC) region. As shown for pAtK84b (Oger and Farrand 2002), nopaline probably induces the conjugal transfer of this plasmid.

### Evolution of pTiC5.7/pTiC6.5 and Their Relationships with Related Ti Plasmids

Most of the regions of pTiC5.7/pTiC6.5 were highly homologous to the corresponding regions of the pTiC58, and pTi-SAKURA (fig. 2*A*). For instance, ACC/ARC and VIR regions, including most of the sequence of the T-DNA and accessory region between the REP and TRA regions, were highly conserved between pTiC5.7/pTiC6.5, and pTiC58. Overall, comparative genomic analysis indicated that pTiC5.7/pTiC6.5, pTiC58, and pTi-SAKURA originate from a common ancestor, although they have diverged during evolution. These plasmids carried almost identical sets of TE, with the exception of pTi-SAKURA, which carried an additional TE (IS1182 family) within the accessory region between REP and TRA regions. This accessory region of pTi-SAKURA was different from the corresponding region in other plasmids used for comparison and is most likely created by horizontal gene transfer (HGT; fig. 2*A*). Although homologous, regions NOC, TRB and REP of pTiC5.7/pTiC6.5 were more distantly related to those of pTiC58 and pTi-SAKURA (86–94% ANI). These regions possibly diverged considerably during evolution. Alternatively, they could have a different origin and might have been introduced by recombination. In this respect, DNA stretch containing contiguous regions NOC, TRB, and REP, and a large portion of accessory region in pTiC5.7/pTiC6.5 was almost identical as corresponding region of pAtK84b (see below section “Evolutionary Relationships and Classification of Ti and OC Plasmids”).

Furthermore, phylogenetic analysis (supplementary fig. S5, Supplementary Material online) and whole plasmid comparisons (fig. 2*A*) suggested that a part of the TRA region of pTiC58 containing *traG*, *traD*, *traC*, and *traA* genes was significantly different compared with the corresponding region in pTiC5.7/pTiC6.5 and pTi-SAKURA. As suggested by Wetzel et al. (2015) and our results, these differences might be the consequence of a recombination event. Overall, although some similarities can be observed, phylogenetic trees based on single genes of plasmid backbone REP, TRA and TRB regions, including QS regulatory genes *traM* and *traI*, were generally incongruent (supplementary figs. S1, S5, S6, and S7, Supplementary Material online). Different tree topologies and clustering of plasmids sequenced in this study were observed both between single gene trees referring to the same backbone region, as well as between trees corresponding to different regions. These results might indicate that REP, TRA and TRB backbone regions did not coevolve, but also suggest the existence of intra-operon recombination in the evolution of Ti and OC plasmids studied.

Plasmids pTiC5.7/pTiC6.5 had similar organization of T-DNA compared with pTiC58 and pTi-SAKURA (fig. 4). Their left parts comprising genes *a*-to-*acs* were highly homologous, showing nucleotide identity >99%. Nevertheless, pTiC5.7/pTiC6.5 had different organizations of the right part of the T-DNA. Unlike pTiC58 and pTi-SAKURA, it carried gene *3′*, whereas the gene *6a* was absent. Moreover, the gene *6b* in pTiC5.7/pTiC6.5 was distantly related to the *6b* gene in pTiC58/pTi-SAKURA, sharing just ∼62% nucleotide identity. Genes *3*′, *6a* and *6b* belong to the so-called group of phenotypic plasticity (*plast*) genes that can be involved in crown gall tumorigenesis, although the molecular basis of their activity has not been fully understood thus far (for a review, see Britton et al. 2008; Otten 2018).

Our results suggested that the T-DNA of pTiC5.7/pTiC6.5 resembles the organization of pTi82.139 (Drevet et al. 1994; Otten et al. 2008). It has been hypothesized that pTi82.139 T-DNA is most likely derived from a pTiC58-like T-DNA, in which the *6a*-*6b* fragment is replaced with a distinct *6b*-*3′* gene fragment (Drevet et al. 1994; Otten and De Ruffray 1994). Indeed, in the case of pTiC5.7/pTiC6.5 T-DNA, we found a probable junction between pTiC58-like and a sequence located at the right part of the T-DNA. In this respect, gene *5* showed a mosaic-like structure, with the left part exhibiting high nucleotide identity (97.4%) to the corresponding gene fragment of pTiC58, whereas the right part was distinct and showed high nucleotide identity (97.6%) to the corresponding gene region of pTiAB4 carried by *All. vitis* strain AB4. The adjacent genes on the left side of pTiC5.7/pTiC6.5 T-DNA (*iaaH*-to-*nos*) also showed a high degree of nucleotide identity (∼95–99%) to the corresponding T-DNA genes in pTiAB4. However, the structure of the pTiAB4 T-DNA is itself chimeric (Otten and De Ruffray 1994). Although its left part (*acs*-*iaaH*) is highly homologous to the left border region of the octopine/cucumopine (o/c) Ti plasmid T-DNA, such as pTiTm4 (99–100% nucleotide identity; Acc. No. U83987), the right part comprising genes *iaaM* to *nos* was highly homologous to corresponding T-DNA genes of pTi82.139 (Otten and De Ruffray 1994) or pTiC5.7/pTiC6.5 (96.1–99.2% nucleotide identity). Therefore, it is assumed that pTiAB4 is formed by HGT between different Ti plasmids, including at least pTi82.139- and o/c Ti plasmid-like structures (Otten and De Ruffray 1994).

### Plasmids pOC-Colt5.8 and pAtK84b Had a Different Evolutionary History

Plasmid pOC-Colt5.8 was most closely related to pAtK84b, as suggested by ANI (95.66%; ∼79% of sequence aligned) and whole plasmid sequence comparison (fig. 2*B*). Although large accessory regions in these plasmids were highly conserved (e.g., NOC, ACC and ARC), their backbone regions were more distantly related. Moreover, plasmids pOC-Colt5.8 and pAtK84b carried distinct sets of TE, suggesting that they had a different evolutionary history. However, it cannot be excluded that these plasmids share a more distant common ancestor.

### Evolutionary Relationships and Classification of Ti and OC Plasmids

In general, data regarding evolutionary relationships between Ti and OC plasmids are limited. Although Clare et al. (1990) suggested that pAtK84b arose as a deletion derivative of the pTiC58-like plasmid, this hypothesis is unlikely considering the distinct organization and lack of homology among some functional regions in these plasmids, as noted by Oger and Farrand (2002). Similarly, comparative genomic analysis performed in this study excludes such scenarios in plasmids pTiC5.7/pTiC6.5 and pOC-Colt5.8. Although some of their regions were highly conserved, such as large gene clusters between REP and TRA regions, most genetic elements in pTiC5.7/pTiC6.5 and pOC-Colt5.8 showed weak or no homology (fig. 2). Taken together, the results obtained suggest a large evolutionary distance between the nopaline-type Ti and OC plasmids characterized in this study, although they might have a common ancestor. Consistent with this, they carried distinct sets of TE (figs. 1 and 2; supplementary table S2, Supplementary Material online).

Furthermore, although plasmids pTiC5.7/pTiC6.5 showed highest level of ANI to the pTiC58 (96.97%; >93% of aligned sequence for both plasmids), some of their genetic elements were more similar to the pAtK84b. Thus, contiguous regions NOC, TRB, REP and a large portion of accessory region were highly conserved (>99% ANI; see also fig. 2*A*) between pTiC5.7/pTiC6.5 and pAtK84b, suggesting the common origin of this large DNA stretch. In other words, these plasmids might have common origin, but it cannot be excluded that this common DNA stretch was created by cointegration between different plasmids or by HGT. On the other hand, although it showed high homology to this region, plasmid pTiC58 possessed a distinct TRB region (∼86% ANI). Most likely, this region has a different origin and might have been introduced by recombination.

Interestingly, pTiC5.7/pTiC6.5, pTiC58 and pTi-SAKURA carried a specific genetic module (“ACC”) between VIR and T-DNA regions (fig. 2*A*) that showed homology to several genes contained in the ACC region of OC plasmids pOC-Colt5.8 and pAtK84b. ORFs located within this DNA stretch were more distantly related or unrelated to putative genes of the ACC region in Ti plasmids pTiC5.7/pTiC6.5, pTiC58 and pTi-SAKURA. Moreover, our analysis suggested that most ORFs within the “ACC” region might produce truncated and most likely nonfunctional proteins. Therefore, a DNA stretch between the VIR and T-DNA regions in nopaline-type Ti plasmids might be a remnant originating from a pOC-Colt5.8/pAtK84b-like ACC structure. This pOC-Colt5.8/pAtK84b-like ACC structure could have been affected by the introduction of various insertion elements, among which some could have contained elements of “proto T-DNA.” It could therefore be hypothesized that some of these pOC-Colt5.8/pAtK84b-like ACC elements were involved in the construction of a “proto T-DNA.” In this regard, considering their relatedness, it has been previously suggested that genes *acs* (agrocinopine synthase) and *accF* (agrocinopine phosphodiesterase) have evolved from a common ancestor (Kim and Farrand 1997). Additionally, it has been indicated that T-DNA biosynthesis genes might originate from degradative opine genes (reviewed in Dessaux et al. 1998; Dessaux and Faure 2018b). Indeed, ACC-like sequences between the VIR and T-DNA regions are adjacent to the T-DNA gene *a*, which is homologous to the *acs* gene. Consistent with this, the nopaline synthesis (*nos*) gene adjoins the nopaline catabolic (NOC) region and exhibits relatedness to the gene encoding opine dehydrogenase (*odh*) at the amino acid level (Kuzmanović, unpublished data).

The ACC/ARC region showed distinct organization in Ti and OC plasmids (figs. 2 and 6*B*). The ACC region in nopaline-type Ti plasmids is structurally and functionally different with respect to the corresponding region in nopaline-type OC plasmids (Hayman and Farrand 1990); it probably evolved independently or was acquired from another plasmid through HGT. On the basis of comparative genomic analysis, the ACC/ARC region of pTiC5.7/pTiC6.5 and pOC-Colt5.8 were classified as pTiC58/pTi-SAKURA-like and pAtK84b-like, respectively (table 2). In general, ACC regions show considerable diversity among different agrobacteria. On the basis of Southern hybridization analysis, at least four different classes of ACC regions were distinguished, exemplified by plasmids pTiC58 (Class I), pArA4a (Class II), pTiBo542 (Class III) and pAtK84b (Class IV; Hayman and Farrand 1990). Additionally, sequence analysis suggested the existence of one more class represented by the plasmid pF5.1b (Kuzmanović and Puławska, unpublished data). On the other hand, the pTiC58-like and pAtK84b-like ARC regions showed some structural similarities, suggesting that they have evolved from a common ancestral operon (Oger and Farrand 2002).

The VIR region in Ti plasmids was likely also acquired by HGT, for example, through cointegration with another replicon. T-DNA transfer (*vir*) and plasmid conjugation systems are related and probably evolved from a common ancestor (reviewed in Christie 1997). Taken together, although speculative, it is tempting to hypothesize that the nopaline-type Ti plasmid originates from the nopaline-type OC plasmid. Consistent with this hypothesis, large regions were highly conserved within Ti and OC plasmids (fig. 2).

Nevertheless, the reconstruction of the evolutionary history of Ti and OC plasmids is still presumptive. Ti plasmids evolved through extensive HGT (Otten et al. 1992; Otten and De Ruffray 1994; Otten et al. 2008). In other words, their evolution was driven by large-scale events, such as recombinations, deletions or insertions, rather than mutations, which was also suggested by this study. The same certainly applies to genetically similar OC plasmids. Therefore, additional data that include important evolutionary intermediates are required to further support this evolutionary model.

As seen in the data presented above, Ti and OC plasmids have a “patchwork” structure composed of diverse genetic elements that makes their classification difficult. In any case, in this study, we proposed a classification scheme that is based on separate comparative analysis of each functional element of the plasmid studied. Each functional element was classified based on comparison with the corresponding element of agrobacterial plasmids characterized so far (table 2).

### OC Plasmids Are Widespread

Occurrence and distribution of OC plasmids associated with crown gall tumors were not extensively studied. PCR analysis performed in this study suggested that the majority of nontumorigenic *Rhizobiaceae* strains studied carry OC plasmids with genes involved in the catabolism of nopaline. It seems that OC plasmids are widespread in different geographical areas and closely associated with crown gall tumors, although their ecological role remains to be studied.

## Conclusion

Tumorigenic strains C5.7 and C6.5 carrying Ti plasmids (pTiC5.7/pTiC6.5), and nonpathogenic strain Colt5.8 carrying the OC plasmid pOC-Colt5.8 were associated with the same crown gall tumor. Plasmids pTiC5.7/pTiC6.5 and pOC-Colt5.8 belonged to the *repABC* family of plasmids and carried a complete set of putative conjugal transfer genes, including QS regulatory genes. They carried putative gene clusters responsible for the catabolism of opines nopaline and agrocinopine and were classified as nopaline-type Ti plasmids, following the traditional opine-based classification. Whole plasmid sequence analysis indicated that pTiC5.7/pTiC6.5 and related nopaline-type Ti plasmids described before (pTiC58 and pTi-SAKURA) originate from a common ancestor, although they have diverged during evolution. However, comparative genomics analysis revealed that pTiC5.7/pTiC6.5 and pAtK84b possesses a large common contiguous region that includes NOC, TRB, REP and a large portion of accessory genes. Most likely, this large DNA stretch has a common origin, suggesting evolutionary relationship between Ti and OC plasmids. Plasmid pOC-Colt5.8 was most closely related to the well-known OC plasmid pAtK84b, although analysis suggested that they had different evolutionary histories and seem to share a more distant common ancestor. Overall, we hypothesized that nopaline-type Ti plasmid might originate from the nopaline-type OC plasmid. Nevertheless, the reconstruction of the evolutionary history of Ti and OC plasmids is still speculative and more data are required in order to support this hypothesis. Furthermore, our results suggested that OC plasmids are widespread and closely associated with crown gall tumors, and definitely deserve more attention in studies referring to ecology of crown gall disease. Finally, we proposed a thorough scheme for classification of Ti and OC plasmids that is based on separate comparative analysis of each functional element of the plasmid studied.

## Supplementary Material

Supplementary data are available at *Genome Biology and Evolution* online.

## Supplementary Material

Supplementary_Material_evz091

## Notes

**Data deposition:** The whole plasmid sequences obtained in this study have been deposited at GenBank (NCBI) under the following accession numbers: MF511177 (pTiC5.7), MK318973 (pOC-Colt5.8), and MK318986 (pTiC6.5)
