# Supplementary material for: Evolutionary Relatedness and Classification of Tumor-Inducing and Opine-Catabolic Plasmids in Three Rhizobium rhizogenes Strains Isolated from the Same Crown Gall Tumor
Source: Genome Biol Evol. 2019 Apr 27;11(6):1525–40. doi: 10.1093/gbe/evz091 (PMC6546132; doi:10.1093/gbe/evz091)
Supplement: Supplementary_Material_evz091 [file supplementary_material_evz091.zip › Supplementary_Figures.pdf]

A. RepA (Fig. S1)

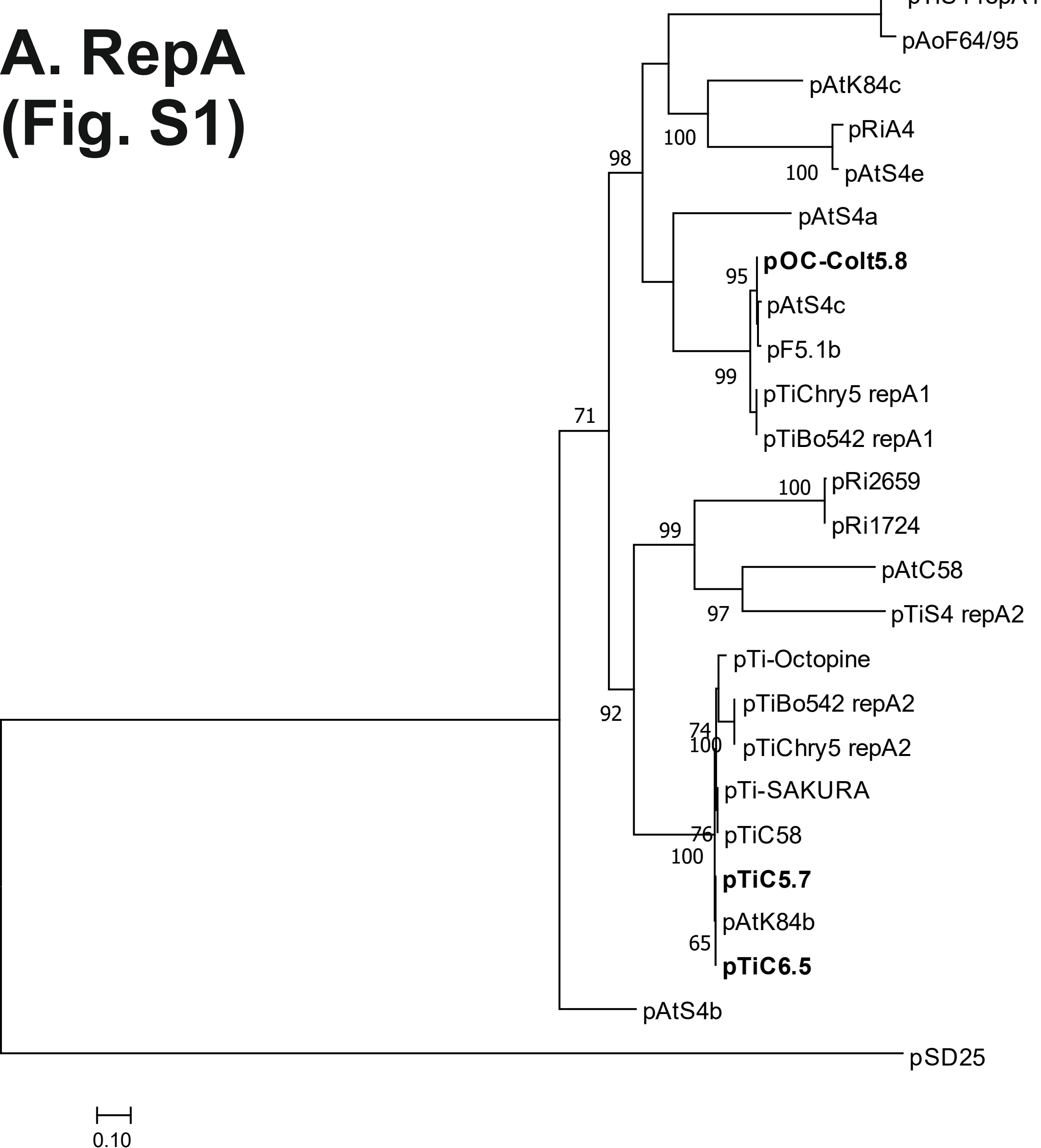

pTiS4 repA1
pAoF64/95
pAtK84c
100
pRiA4
100
pAtS4e
98
pAtS4a
pOC-Colt5.8
95
pAtS4c
pF5.1b
99
pTiChry5 repA1
71
pTiBo542 repA1
100
pRi2659
pRi1724
99
pAtC58
97
pTiS4 repA2
pTi-Octopine
pTiBo542 repA2
92
74
100
pTiChry5 repA2
pTi-SAKURA
76
pTiC58
100
pTiC5.7
pAtK84b
65
pTiC6.5
pAtS4b
pSD25
0.10

B. RepB (Fig. S1)

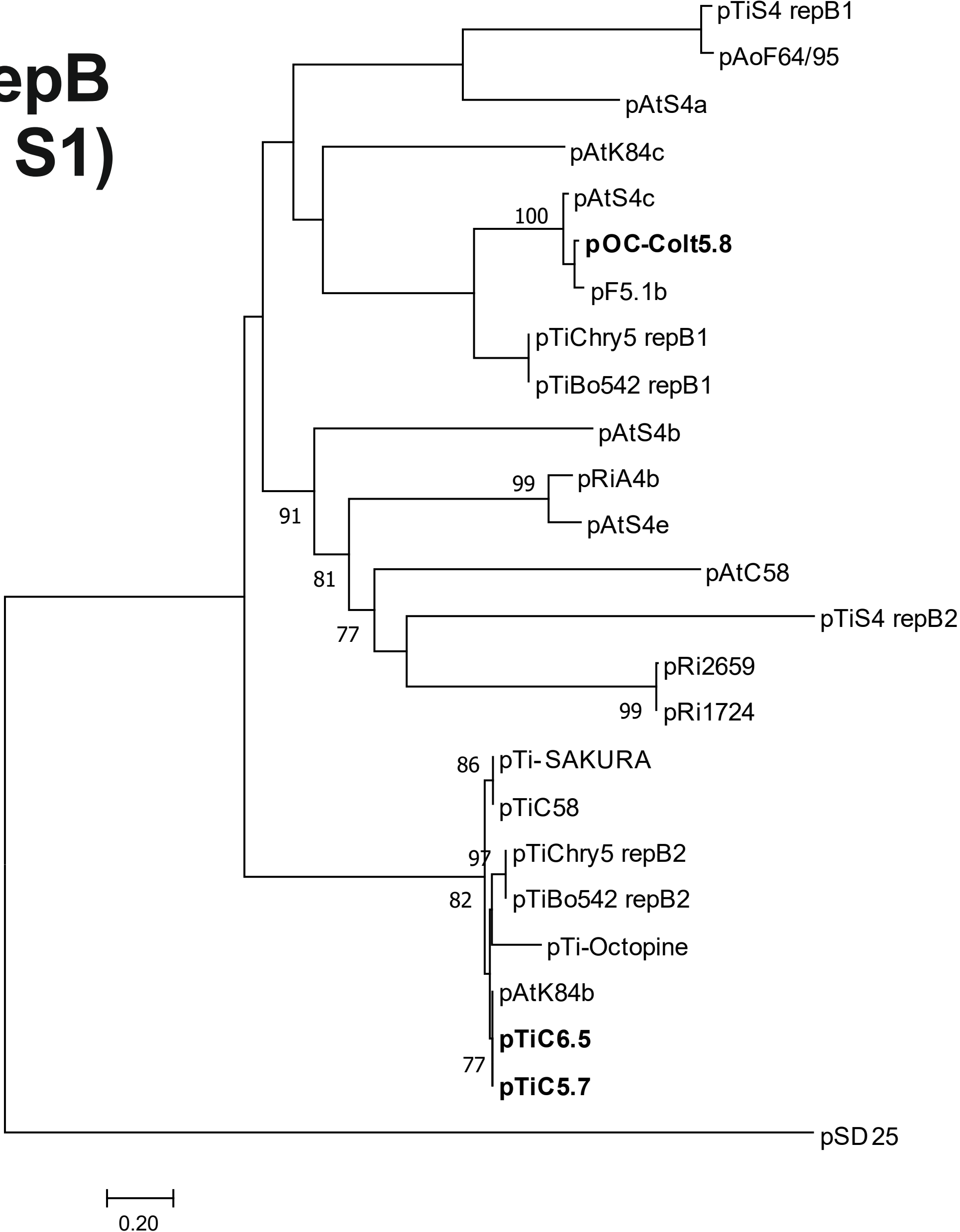

pTiS4 repB1
pAoF64/95
pAtS4a
pAtK84c
pAtS4c
100
pOC-Colt5.8
pF5.1b
pTiChry5 repB1
pTiBo542 repB1
pAtS4b
99
pRiA4b
pAtS4e
91
81
pAtC58
77
pTiS4 repB2
pRi2659
99
pRi1724
86
pTi-SAKURA
pTiC58
97
pTiChry5 repB2
82
pTiBo542 repB2
pTi-Octopine
pAtK84b
pTiC6.5
77
pTiC5.7
pSD 25
0.20

C. RepC (Fig. S1)

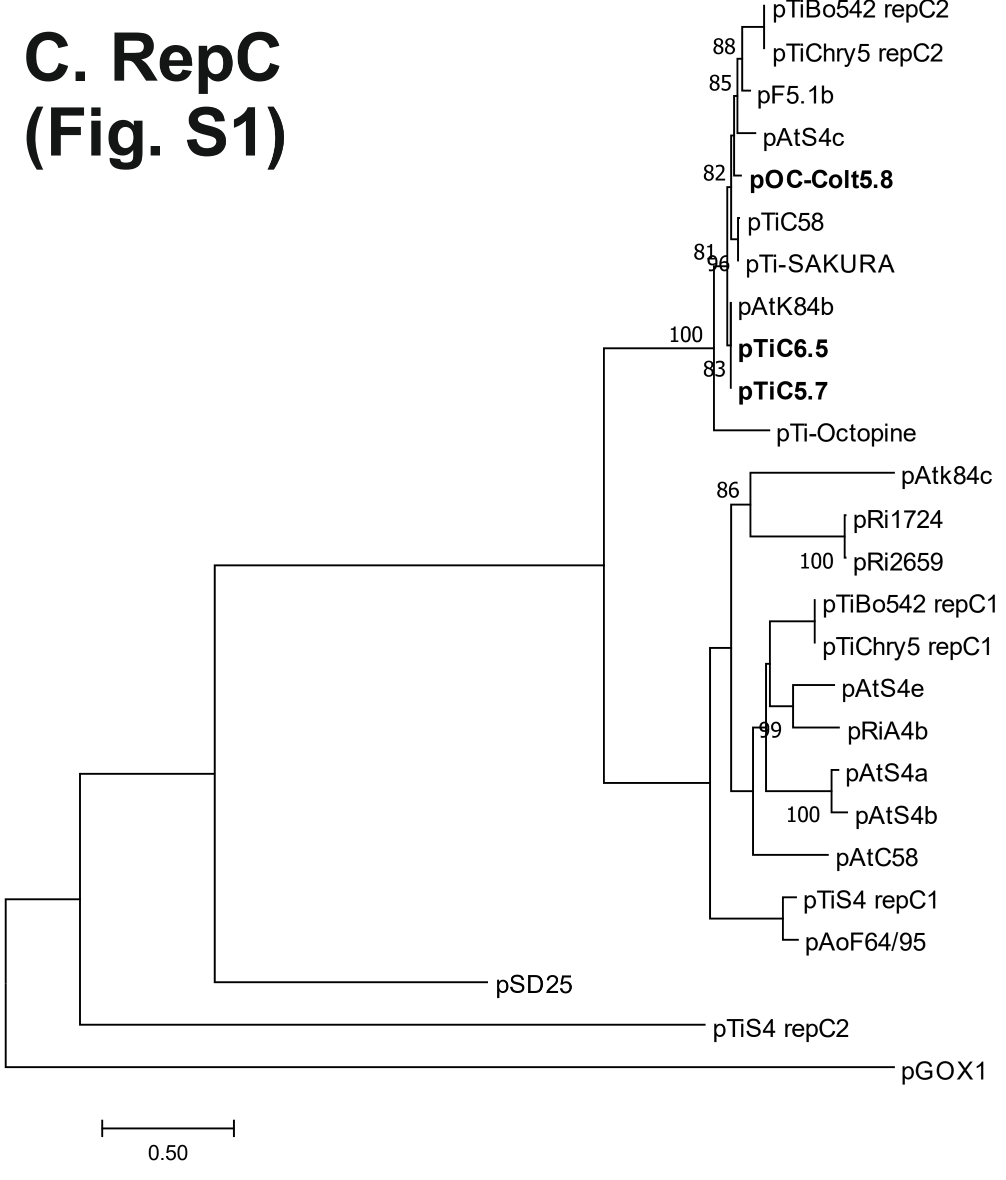

Fig. S1. Maximum likelihood trees based on RepA (A), RepB (B) and RepC (C) protein sequences indicate the phylogenetic position of plasmids studied (marked in bold) and their relationships with related plasmids. The trees were constructed using models LG+G+F (A and C) and LG+G+I+F (B). Branch support values (expressed as percentages) estimated by the aLRT SH-like method are shown at the nodes (values equal to or higher than 60 % are shown). The scale bar represents the estimated number of amino acid substitutions per site. Plasmids pSD25 (A and B) and pGOX1 (C) were used as outgroups. DDBJ/EMBL/GenBank accession numbers of plasmids included into analysis are shown in Table S3.

```
pTiC5.7/pTiC6.5    CCAAGGGCGCAATTATACGTCGCTGGCGCGACGCGTTG
pOC-Colt5.8        CCAAGGGCGCAATTATACGTCGCTGACGCGACGCCTTG
pTiC58             CCAAGGGCGCAATTATACGTCGCTGACGCGACGCCTTG
                   ************************.********* ***
```

Fig. S2 The nucleotide sequence alignment of the *oriT* region of plasmids pTiC5.7/pTiC6.5, pOC-Colt5.8 and pTiC58. The asterisk at the bottom line of the alignment indicates identical (fully conserved or invariant) bases, whereas single dot indicates highly conserved bases.

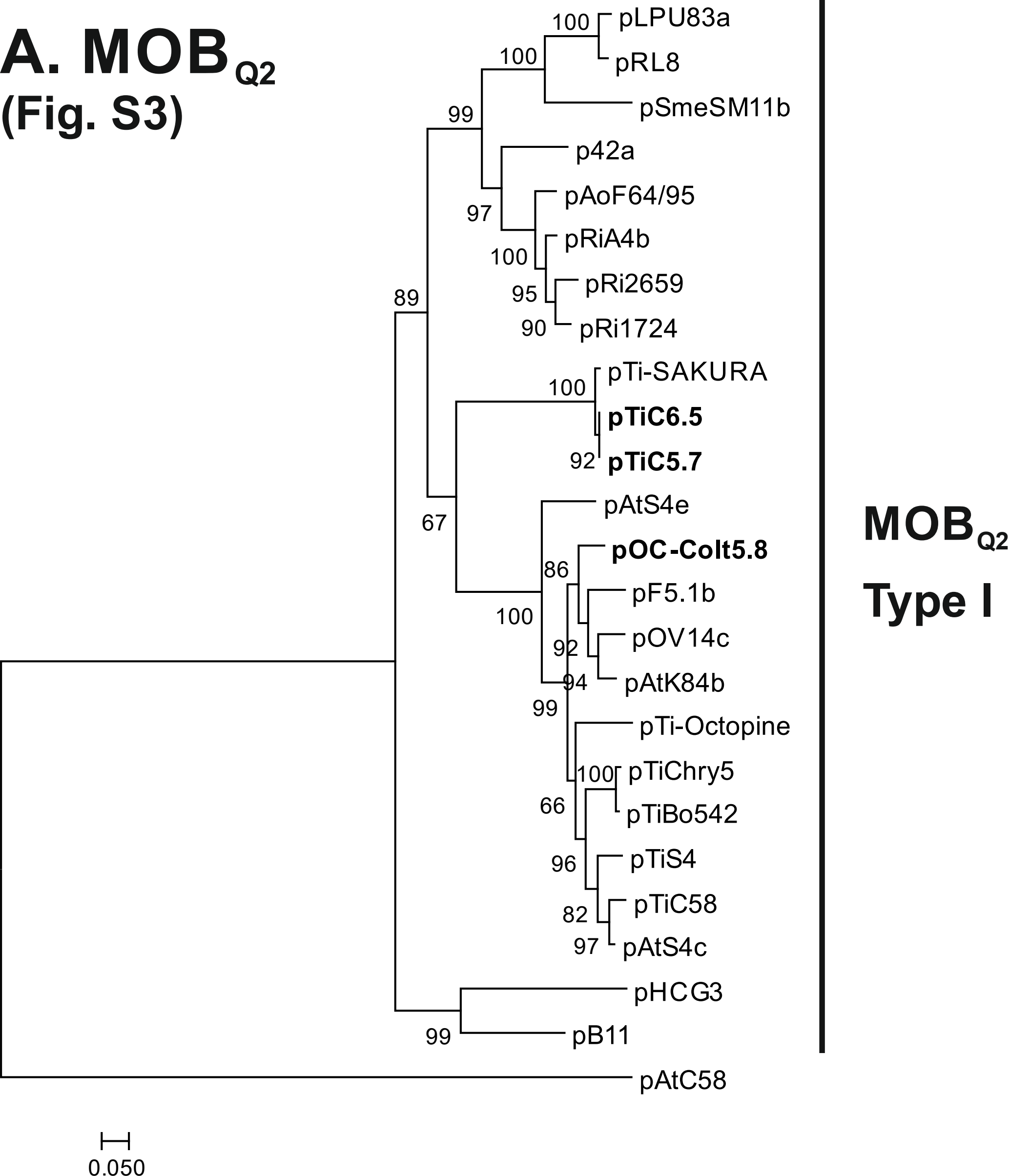
A. MOB$_{Q2}$
(Fig. S3)
pLPU83a
pRL8
pSmeSM11b
p42a
pAoF64/95
pRiA4b
pRi2659
pRi1724
pTi-SAKURA
pTiC6.5
pTiC5.7
pAtS4e
pOC-Colt5.8
pF5.1b
pOV14c
pAtK84b
pTi-Octopine
pTiChry5
pTiBo542
pTiS4
pTiC58
pAtS4c
pHCG3
pB11
pAtC58
100
100
99
97
100
95
90
89
100
92
67
86
100
92
94
99
100
66
96
82
97
99
MOB$_{Q2}$
Type I
0.050

B. MOB_{P2} (Fig. S3)

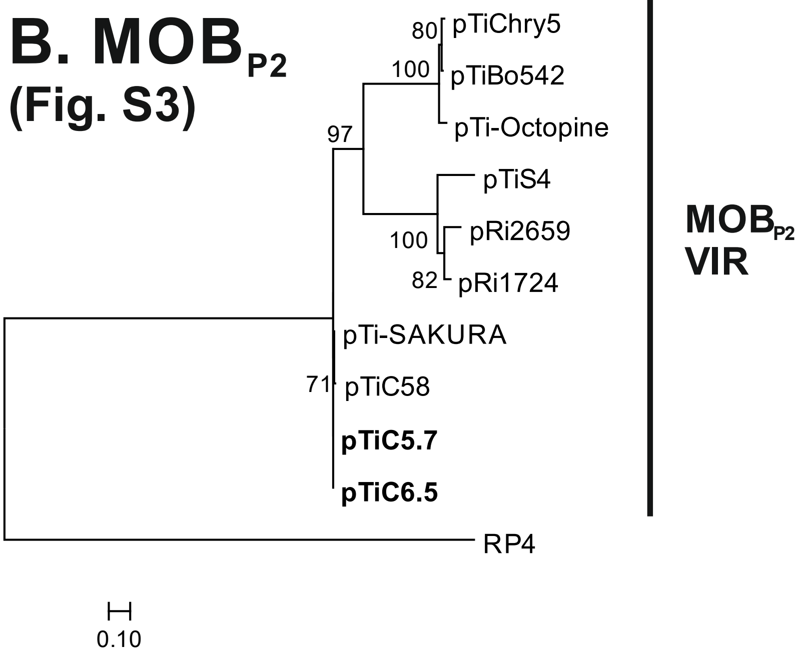

Fig. S3. Maximum likelihood trees based on protein sequences of conjugative relaxases TraA (A) and VirD2 (B) indicate the phylogenetic position of plasmids studied (marked in bold) and their clustering into the MOB families MOB_{Q2} (A) and MOB_{P2} (B). The trees were constructed using models LG+G+I+F (A) and JTT +G+F (B). Branch support values (expressed as percentages) estimated by the aLRT SH-like method are shown at the nodes (values equal to or higher than 60 % are shown). The scale bar represents the estimated number of amino acid substitutions per site. Plasmids pAtC58 (A) and RP4 (B) were used as outgroups. The names of the plasmid MOB families and groups discussed in the text are shown at the right of each tree. DDBJ/EMBL/GenBank accession numbers of plasmids included into analysis are shown in Table S3.

A. T4SS_MOB$_{Q2}$
(Fig. S4)

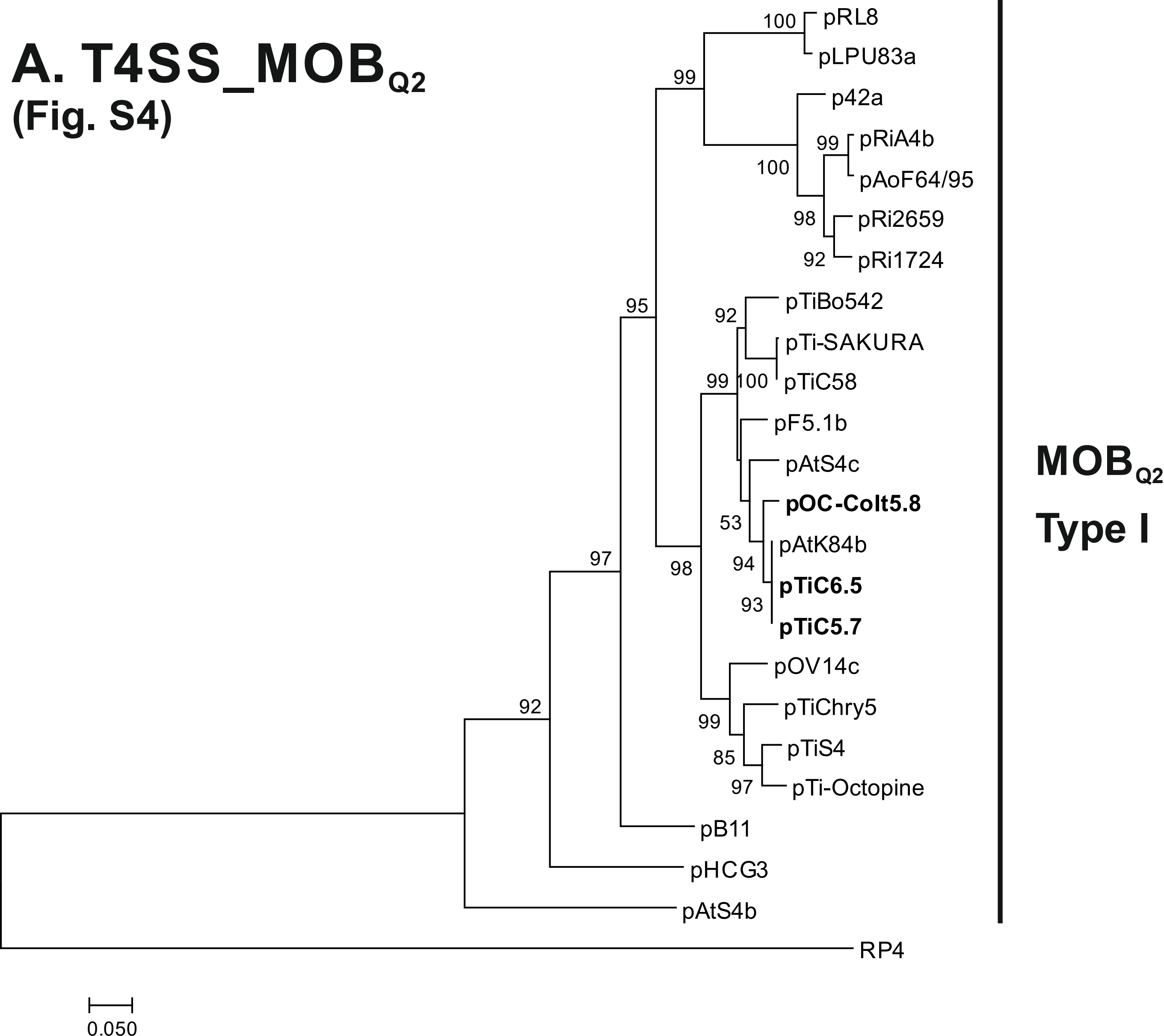

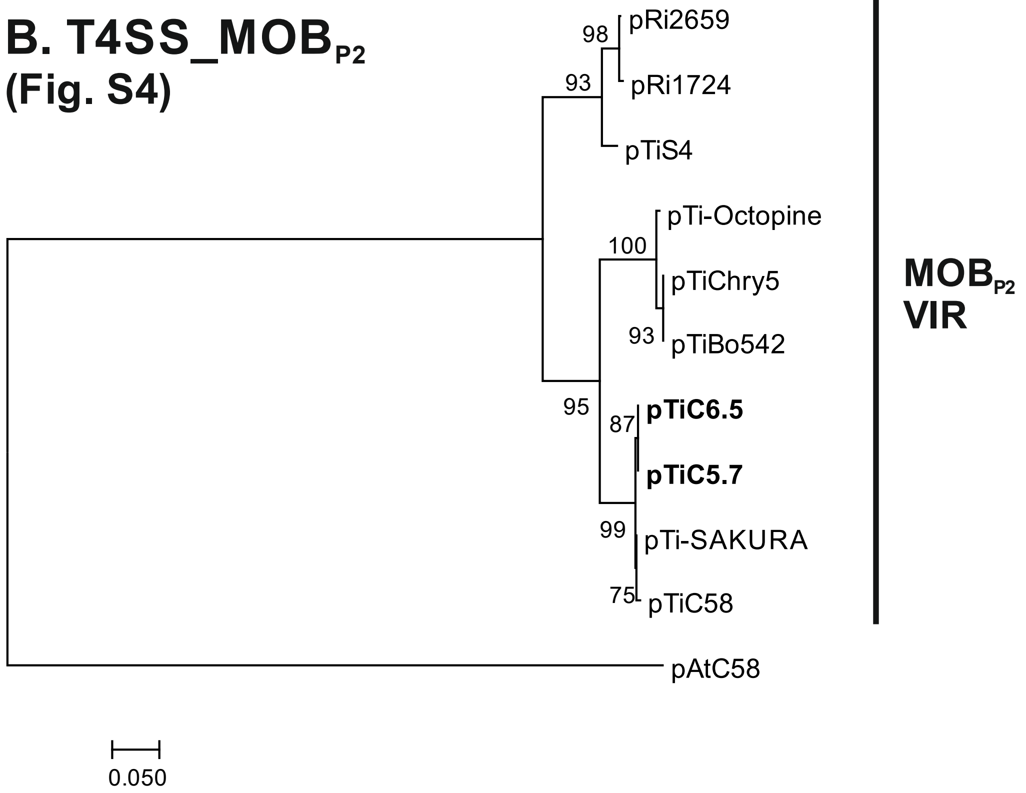

Fig. S4. Maximum likelihood trees based on T4SS TrbE (A) and VirB4 (B) protein sequences indicate the phylogenetic position of plasmids studied (marked in bold) and their relationship with related plasmids. The trees were constructed using model LG+G+F. Branch support values (expressed as percentages) estimated by the aLRT SH-like method are shown at the nodes (values equal to or higher than 60 % are shown). The scale bar represents the estimated number of amino acid substitutions per site. Plasmids RP4 (A) and pAtC58 (B) were used as outgroups. The names of the plasmid MOB families and groups discussed in the text are shown at the right of the tree. DDBJ/EMBL/GenBank accession numbers of plasmids included into analysis are shown in Table S3.

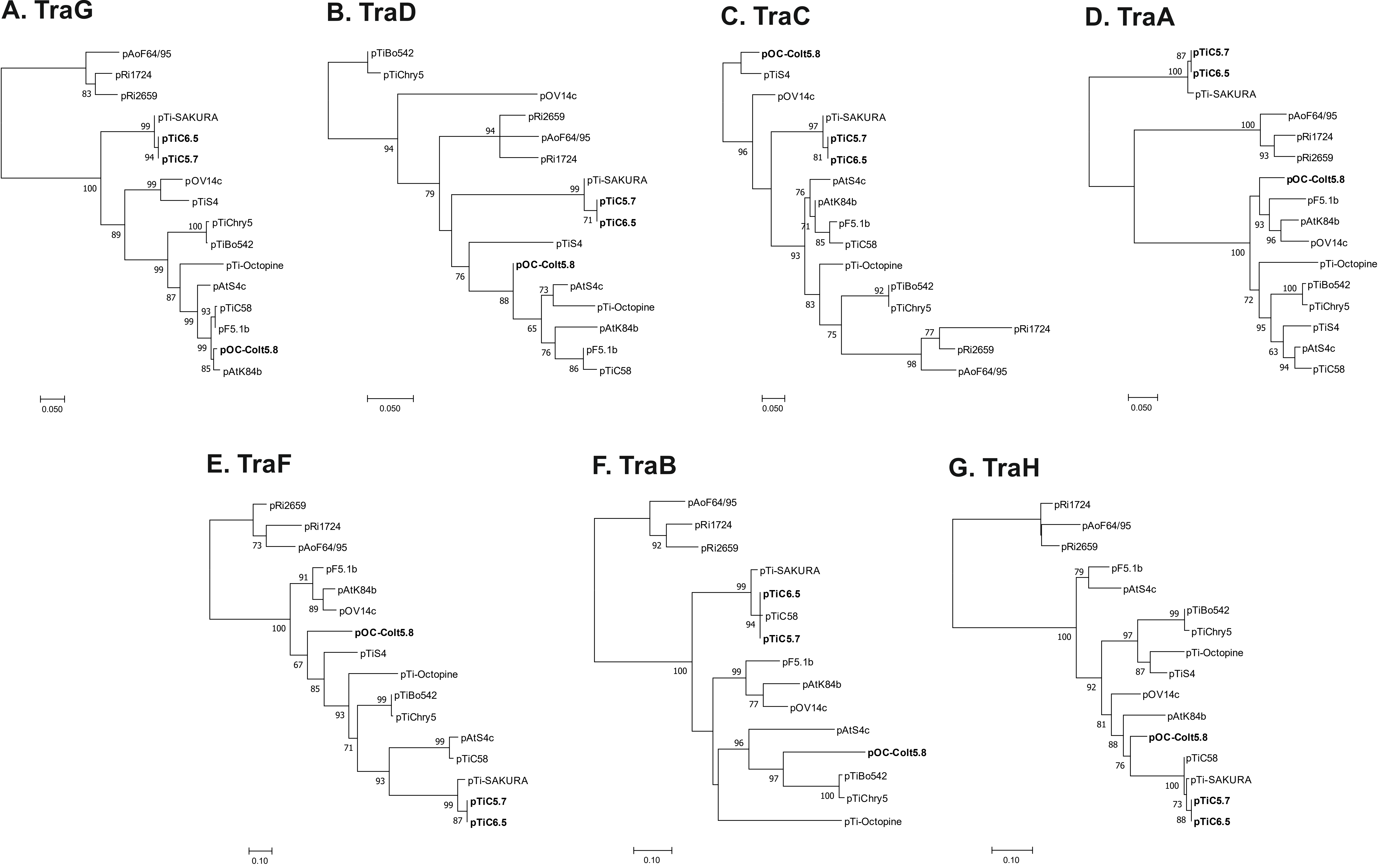

Fig. S5. Maximum likelihood trees based on TraG (A), TraD (B), TraC (C), TraA (D), TraF (E), TraB (F) and TraH (G) protein sequences indicate the phylogenetic position of plasmids studied (marked in bold) and their relationships with related plasmids. The trees were constructed using models JTT+G+F (A, C and D), JTT+G (B and G), WAG+G+F (E) and CpREV+G+F (F). Branch support values (expressed as percentages) estimated by the aLRT SH-like method are shown at the nodes (values equal to or higher than 60 % are shown). The scale bar represents the estimated number of amino acid substitutions per site. DDBJ/EMBL/GenBank accession numbers of plasmids included into analysis are shown in Table S3.

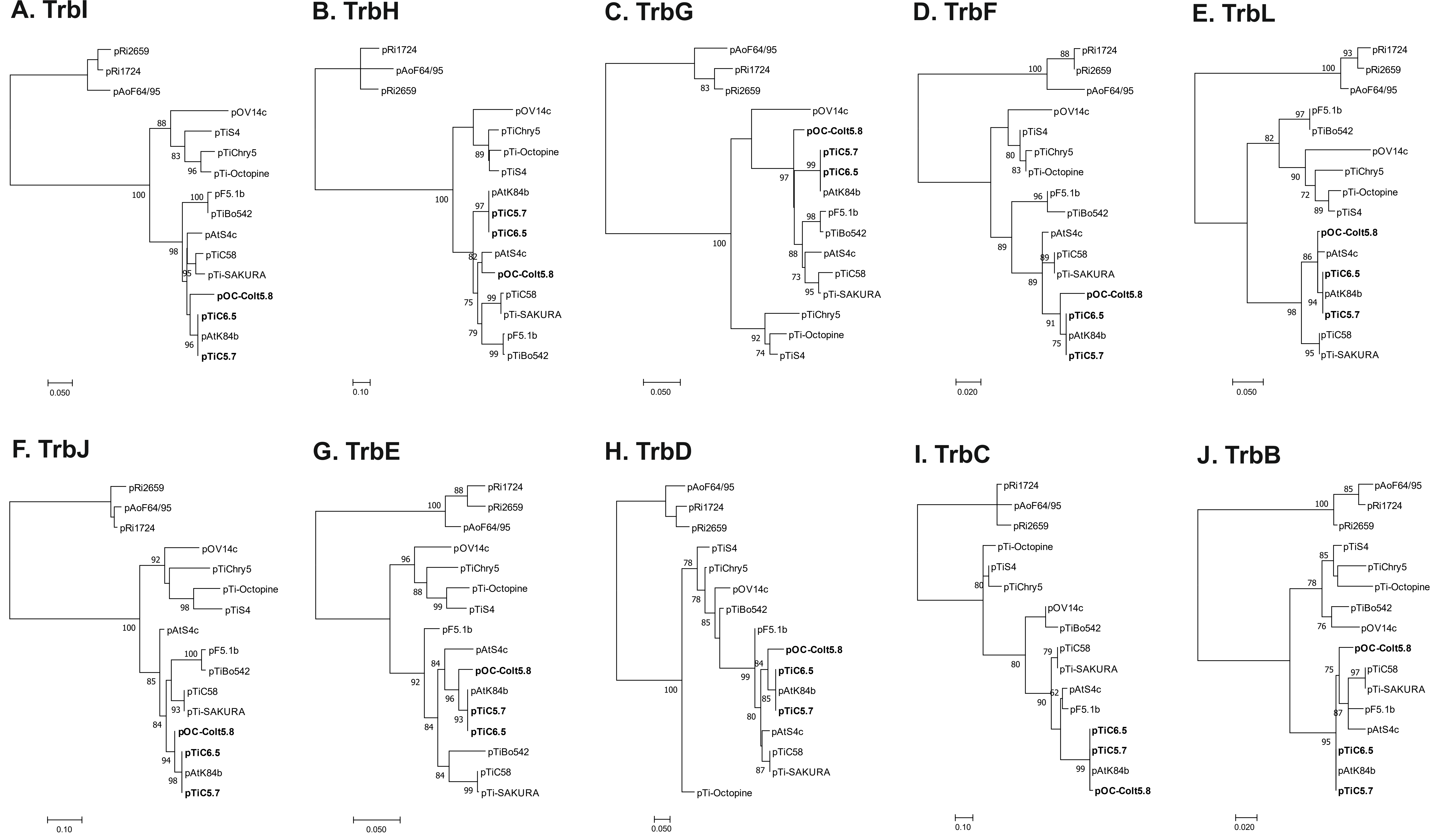

Fig. S6. Maximum likelihood trees based on TrbI (A), TrbH (B), TrbG (C), TrbF (D), TrbL (E), TrbJ (F), TrbE (G), TrbD (H), TrbC (I) and TrbB (J) protein sequences indicate the phylogenetic position of plasmids studied (marked in bold) and their relationships with related plasmids. The trees were constructed using models LG+G+F (A and E), HIVb+G+F (B), JTT+G (C and I), MtZoa+G+F (D), HIVb+G+I+F (F), LG+G+I+F (G), LG+G (H) and JTT+G+F (J). Branch support values (expressed as percentages) estimated by the aLRT SH-like method are shown at the nodes (values equal to or higher than 60 % are shown). The scale bar represents the estimated number of amino acid substitutions per site. DDBJ/EMBL/GenBank accession numbers of plasmids included into analysis are shown in Table S3.

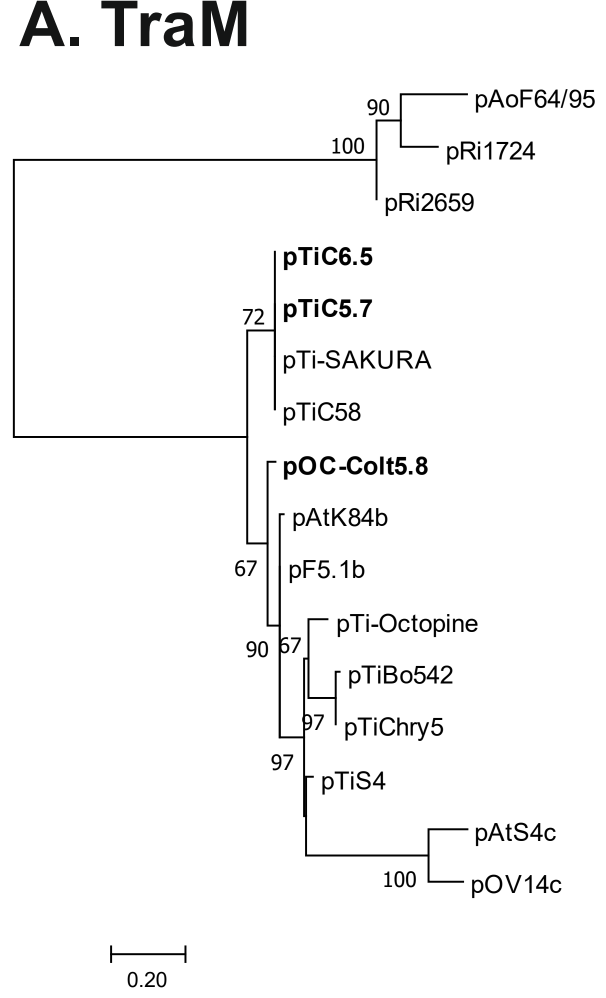

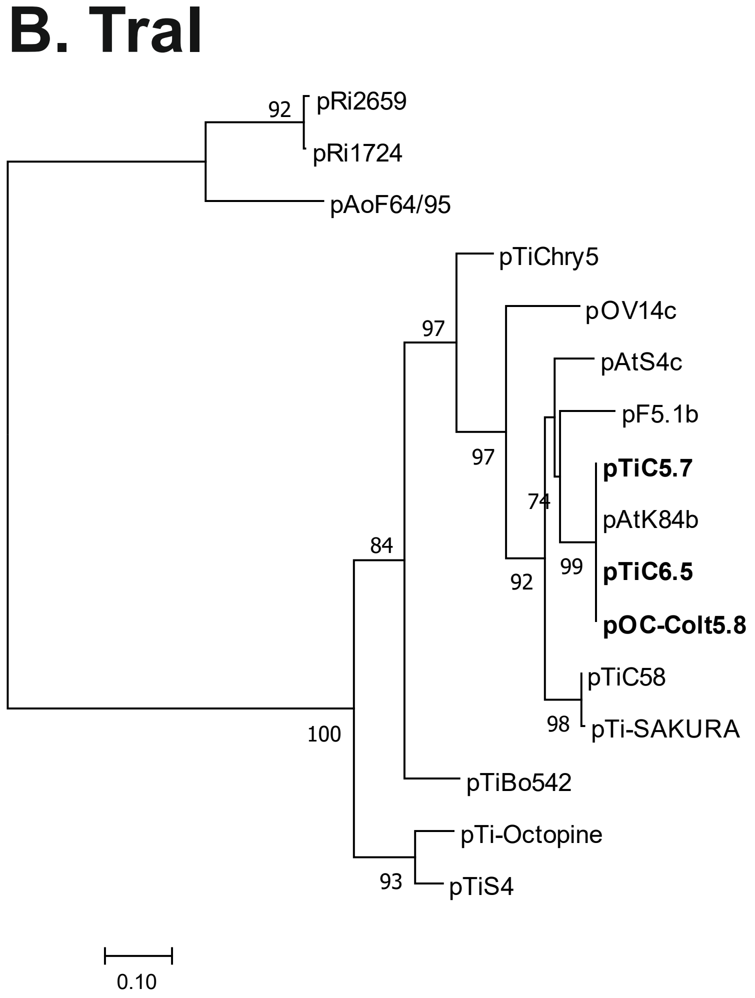

Fig. S7. Maximum likelihood trees based on TraM(A) and TraI (B) protein sequences indicate the phylogenetic position of plasmids studied (marked in bold) and their relationships with related plasmids. The trees were constructed using model JTT+G. Branch support values (expressed as percentages) estimated by the aLRT SH-like method are shown at the nodes (values equal to or higher than 60 % are shown). The scale bar represents the estimated number of amino acid substitutions per site. DDBJ/EMBL/GenBank accession numbers of plasmids included into analysis are shown in Table S3.
